# SAXS analysis of the tRNA-modifying enzyme complex MnmE/MnmG reveals a novel interaction mode and GTP-induced oligomerization

**DOI:** 10.1093/nar/gku213

**Published:** 2014-03-14

**Authors:** Marcus Fislage, Elke Brosens, Egon Deyaert, Alessandro Spilotros, Els Pardon, Remy Loris, Jan Steyaert, Abel Garcia-Pino, Wim Versées

**Affiliations:** 1 Structural Biology Research Center, VIB, Pleinlaan 2, 1050 Brussel, Belgium; 2 Structural Biology Brussels, Vrije Universiteit Brussel, Pleinlaan 2, 1050 Brussel, Belgium; 3 EMBL Hamburg outstation c/o DESY, Notkestrasse 85, Geb. 25A, 22603 Hamburg, Germany

## Abstract

Transfer ribonucleic acid (tRNA) modifications, especially at the wobble position, are crucial for proper and efficient protein translation. MnmE and MnmG form a protein complex that is implicated in the carboxymethylaminomethyl modification of wobble uridine (cmnm^5^U34) of certain tRNAs. MnmE is a G protein activated by dimerization (GAD), and active guanosine-5'-triphosphate (GTP) hydrolysis is required for the tRNA modification to occur. Although crystal structures of MnmE and MnmG are available, the structure of the MnmE/MnmG complex (MnmEG) and the nature of the nucleotide-induced conformational changes and their relevance for the tRNA modification reaction remain unknown. In this study, we mainly used small-angle X-ray scattering to characterize these conformational changes in solution and to unravel the mode of interaction between MnmE, MnmG and tRNA. In the nucleotide-free state MnmE and MnmG form an unanticipated asymmetric α2β2 complex. Unexpectedly, GTP binding promotes further oligomerization of the MnmEG complex leading to an α4β2 complex. The transition from the α2β2 to the α4β2 complex is fast, reversible and coupled to GTP binding and hydrolysis. We propose a model in which the nucleotide-induced changes in conformation and oligomerization of MnmEG form an integral part of the tRNA modification reaction cycle.

## INTRODUCTION

Transfer ribonucleic acid (tRNA) molecules contain a vast number of modified nucleotides. To date, over 90 of these modifications are known ranging from simple methylations to complex hypermodifications (1,2). Those modifications play structural or functional roles contributing to (i) the proper fold and stability of tRNA, (ii) proper codon–anticodon interaction at the decoding center of the ribosome and (iii) tRNA recognition by the cognate aminoacyltransferase (3). One of the main modification sites of tRNA is position 34, the so-called wobble position, that directly interacts with the third nucleotide of the messenger RNA (mRNA) codon. Considering their role in translation efficiency and fidelity, wobble modifications probably belong to the minimal set of tRNA modifications used in ancestral organisms (4).

In bacteria, the proteins MnmE and MnmG form an enzyme complex (MnmEG) that is implicated in the modification of the wobble uridine in tRNA^Lys^_mnm5s2UUU_, tRNA^Glu^_mnm5s2UUC_, tRNA^Gln^_cmnm5s2UUG_, tRNA^Leu^_cmnm5UmAA_, tRNA^Arg^_mnm5UCU_ and tRNA^Gly^_mnm5UCC_ (5,6). Except for the latter, all these tRNAs are reading A- and G-ending codons in split codon boxes (7,8). Depending on the substrate that is being used, the MnmEG complex first forms either 5-carboxymethylaminomethyluridine (cmnm^5^U-using glycine as substrate) or 5-aminomethyluridine (nm^5^U-using ammonium as substrate) (9). In a later step the bifunctional enzyme MnmC can convert these products to 5-methylaminomethyluridine (mnm^5^U), and finally the sulfur adding enzyme MnmA, in collaboration with a number of other proteins, will add a sulfur at position 2 of certain tRNAs, leading to mnm^5^s^2^U (10,11). tRNA^Leu^_UAA_ is an exception, as it does not get modified by either MnmC or MnmA, but it does get modified by TrmL, leading to the formation of 5-carboxymethlyaminomethyl-2′-O-methyluridine (cmnm^5^Um) (6).

In eukaryotes, the orthologs of MnmE and MnmG are targeted to mitochondria and modify mitochondrial tRNAs (12). Interestingly, in human mitochondria, these orthologs (called GTPBP3 and MTO1, respectively) incorporate during the modification reaction a taurine molecule instead of glycine, leading to 5-taurinomethyl-uridine (τm^5^U) (13,14). In bacteria, MnmE and especially MnmG have been identified as important regulators and determinants of bacterial virulence (15,16). In human, on the other hand, mutations of these enzymes are involved in severe mitochondrial myopathies (MELAS and MERRF) as well as in non-syndromic deafness (17), and the former two diseases are known to be related to deficiencies in τm^5^U tRNA modification (18). Moreover, it has been recently shown that mutations in MTO1 cause hypertrophic cardiomyopathy and lactic acidosis (19).

MnmE (formerly known as TrmE) is a homodimeric protein of about 50-kDa subunits, where each subunit consists of an N-terminal domain, a helical domain and a G domain that is inserted within the helical domain. The N-terminal domain is involved in homodimerization and is responsible for the binding of a tetrahydrofolate (THF) derivative. This THF derivative has been proposed to be a 5,10-methylene-THF (MTHF) that serves as the one carbon donor for the C5 methylene moiety incorporated in uracil (9). MnmE belongs to the family of G proteins activated by nucleotide-dependent dimerization (GAD) (20,21). Compared to canonical small G proteins from the Ras family, GADs such as MnmE show a fast dimerization-dependent GTP hydrolysis rate combined with a low affinity for guanosine-5'-diphosphate (GDP) (22). This makes them independent of guanine nucleotide exchange factors (GEFs) or GTPase activating proteins (GAPs) to cycle between a GTP-bound ‘on state’ and a GDP-bound ‘off state’ (20,21). Crystal structures of MnmE have shown that in the GDP-bound state the nucleotide-binding sites of the G domains are facing each other, but do not interact (22,23). However, crystal structures of the isolated G domains show that these dimerize upon binding of stable GTP analogues or transition state analogues in a K^+^-dependent manner (24). The occurrence of such nucleotide-induced conformational changes in the G domains is further supported in the context of the full-length protein by electron paramagnetic resonance (EPR) measurements (25). The dimerization of the G domains with concomitant reorganization of switch loops and catalytic machinery, together with the binding of a K^+^ ion in the active site to stabilize the negative charges in the transition state, finally leads to hydrolysis of GTP with rate-limiting dissociation of the G domains (26). Previous experiments have shown that, in contrast to the classical G protein paradigm, an active GTP hydrolysis is required for the tRNA modification to occur *in vitro* and *in vivo* (23).

MnmG (formerly known as GidA) is a homodimeric protein consisting of 70-kDa subunits that bind flavin adenine dinucleotide (FAD) and nicotinamide adenine di-nucleotide. Crystal structures of MnmG from several organisms have shown that each MnmG subunit consists of an FAD-binding domain, an insertion domain and a helical domain (27–29). Previous experiments indicated that MnmG is mainly responsible for tRNA binding within the MnmEG complex (28). Furthermore, two conserved cysteine residues in the vicinity of the active site were identified to be crucial for the tRNA modification reaction and are proposed to play a catalytical role (28).

The currently proposed mechanism of the MnmEG-catalyzed tRNA modification reaction implies that the activities of MnmE and MnmG are interdependent and that co-factors from both proteins (MTHF and FAD) are needed simultaneously in the tRNA modification reaction (6,9). We have previously proposed that the large conformational changes of the G domains of MnmE upon GTP binding and hydrolysis are relayed throughout MnmE and MnmG and orchestrate the tRNA modification reaction (23,25). However, awaiting structural information of the MnmEG complex in different nucleotide-bound states, the nature and relevance of these conformational changes remain poorly understood.

Here we used small-angle X-ray scattering (SAXS) to unravel the mode of interaction between MnmE and MnmG in the α2β2 complex (i.e. one MnmE dimer bound to one MnmG dimer). Surprisingly, MnmE and MnmG interact in an asymmetric manner which is distinct from the earlier proposed model. This model is validated using biophysical measurements. Size exclusion chromatography (SEC) experiments as well as SEC coupled to multiangle light scattering (SEC-MALS) and SAXS indicate that MnmE and MnmG form a higher oligomeric state when GTP is bound (α4β2, i.e. two MnmE dimers binding to one MnmG dimer), and this oligomerization appears to be reversible upon GTP hydrolysis. We propose a model in which the nucleotide-induced changes in conformation and oligomerization of MnmEG form an integral part of the tRNA modification reaction cycle.

## MATERIALS AND METHODS

### Protein expression and purification

The open reading frame coding for MnmE from *Escherichia coli* was cloned in a pET20 vector and the open reading frames coding for MnmG from *E. coli* and *Aquifex aeolicus* were cloned in a pET14b and a pET28a vector, respectively, as described previously (27). Point variants of MnmE and MnmG were prepared via the QuikChange mutagenesis protocol from Stratagene. All proteins were expressed in Rosetta(DE3)pLysS and purified as described earlier (27). For preparation of the MnmE–MnmG α2β2 complex, equimolar amounts of MnmE and MnmG were rapidly mixed in the absence of nucleotides. For preparation of the MnmE–MnmG–MnmE α4β2 complex, MnmE was incubated with 1-mM GDP–AlFx (1-mM GDP, 1-mM AlCl_3_ and 10-mM NaF) and subsequently MnmG was added either in equimolar amount or in a 2 MnmE:1 MnmG stoichiometry.

### Generation and purification of MnmG-specific nanobodies

A llama was immunized over a period of 6 weeks with in total 2 mg of purified MnmEG complex. The immunization, library construction and selection have been performed following standard procedures (30) with minor modifications: total RNA was extracted from the peripheral blood lymphocytes (31) and 50 μg of total RNA was used to prepare complementary deoxyribonucleic acid (cDNA) using SuperScript II (Invitrogen) and dN6 random primers, according to the manufacture's instruction. MnmEG-specific phage was eluted from the MnmEG-coated wells with 100-mM triethylamine (pH 10) and used to infect fresh TG1 cells. The MnmEG-coated wells were washed once with Tris-HCl (pH 6.8), several times with phosphate buffered saline, and freshly grown TG1 cells were added to the wells to recover the non-eluted phage. Sequence analysis revealed nine different nanobody families. Enzyme-linked immunosorbent assay confirmed that most of these, including Nb_MnmG_1, recognize MnmG. Finally, all selected nanobody genes were cloned in a pHEN6 vector for expression with a histidine-tag in *E. coli* and nanobodies were purified via standard procedures (30).

### Preparation of tRNA

The genes coding for *E. coli* tRNA^Lys^(UUU) and *A. aeolicus* tRNA^Lys^(UUU) were cloned in a pAsk-Iba vector and the tDNA including the T7 promotor site was amplified via polymerase chain reaction (PCR). tRNA was transcribed from the PCR product using a T7 RNA polymerase kit (Promega) via run off transcription according to the manufacturer's instructions. Unincorporated nucleotides were removed using MicroSpin G-25 columns (GE Healthcare) and tRNA was purified via precipitation using 0.1 volumes of 3-M sodium acetate and 2.5 volumes of 95% ethanol. To ensure folding of the tRNA to its global energy minimum, the tRNA transcript was heated to 368 K and slowly cooled to 288 K in a buffer consisting of 10-mM MgCl_2_, 100-mM KCl and 20-mM Tris-pH 7.5.

### SAXS data collection and analysis

SAXS experiments were performed at the beamlines P12 and X33 of the EMBL in Hamburg (Germany), at the Swing beamline in Soleil (Paris, France) and at the ID 14-3 BioSAXS beamline at the ESRF in Grenoble (France). In the cases where a batch setup was used, scattering intensities were collected at protein concentrations of 8 mg/ml, 4 mg/ml, 2 mg/ml and 1 mg/ml. In the cases where an online high-performance liquid chromatography (HPLC) setup was used, scattering intensities were collected after injection of 50 μl of 8-mg/ml protein or protein complex on a KW404 gel filtration column (Shodex). This allowed us to collect online SAXS data on the elution peak of interest. In case an online stopped-flow setup was used, 32.5-μM MnmE and 32.5-μM MnmG were rapidly mixed in the measuring capillary in a 1:1 ratio, using a Bio-Logic SFM-300 stopped-flow apparatus coupled to the P12 beamline (EMBL, DESY, Hamburg) and data were collected at different time intervals after mixing. As controls, either 32.5-μM MnmE or 32.5-μM MnmG was mixed with buffer. All SAXS measurements were performed in 20-mM Hepes pH 7.5, 150-mM KCl, 5-mM MgCl_2_ and 2-mM dithiothreitol (DTT), either with or without added nucleotides depending on the experiment. Data were processed using PRIMUS (32). The radius of gyration (*R*_g_) was evaluated using the Guinier approximation (33) and also from the entire scattering curve using Porod's law (34), and the distance distribution function *p*(*r*) was calculated using gnom (35). Guinier plots of all SAXS data support monodispersity of the analyzed samples (Supplementary Figure S1). The measured scatter curves were compared with the theoretical scattering curves of the protein models using Crysol (36). *Ab initio* shape reconstructions were performed using 10–20 independent runs of Dammif (37) followed by Damaver (38) to create the final *ab initio* shape. OLIGOMER (39) was used to evaluated the percentage of open and closed MnmE in the GppNHp-bound state. ScÅtter was used to calculated the molecular weights of the proteins and for the calculation of the fitting parameter *R*_SAS_, the small-angle scattering invariant *V*_C_ and the parameter *Q*_R_ (40). The summary of all statistics is given in Supplementary Table S1.

### Protein modeling, docking and refinement

A model of *E. coli* MnmE was prepared by homology modeling starting from *Chlorobium tepidum* MnmE [PDB code 3GEE (25)] using MODELLER (41) after performing sequence alignments with Kalign (42). A model of Nb_MnmG_1 was prepared starting from a canonical nanobody directed against carbonic anhydrase [PDB code 1F2X (43)] using MODELLER. The complenentarity deterimining regions (CDRs) were refined using GROMACS (44). Docking of MnmE to MnmG, tRNA to MnmG and Nb_MnmG_1 to MnmG was performed using PatchDock (45,46). Hereby, the two partners are docked based on shape matching algorithms and the docking solutions are ranked according to a geometric shape complementarity score. Subsequently, the theoretical scattering curves of all 413 docking models were compared to the experimental SAXS curve to filter out the best fitting model (see Supplementary material and methods for a detailed description of the selection of the MnmEG α2β2 complex). Missing residues not seen in the crystal structure were added using MODELLER (41) and the N-terminal tag was added in Coot (47). Models were refined using molecular dynamics simulations in water using the OPLS force field within GROMACS (44). All simulations were done at a constant temperature of 300 K and a constant pressure of 1 atm over a simulation time of 1 ns. Final figures were created in PyMOL (www.pymol.org).

### Isothermal titration calorimetry

Isothermal titration calorimetry (ITC) experiments were performed at 278 K using the MicroCal iTC200 system (GE Healthcare) in a buffer consisting of 20-mM Hepes pH 7.5, 150-mM NaCl, 5-mM MgCl_2_ and 2-mM β-mercaptoethanol (BME). For binding measurements of wild-type MnmE and MnmG, a protein concentration in the cell and syringe of, respectively, 15 μM and 300 μM was used, in the presence of 1-mM FAD. Titrations were performed in both directions with either MnmE or MnmG in the measuring cell. The binding constants of variants of MnmE and MnmG were measured by titration of 700-μM MnmE (in the syringe) into 50-μM MnmG (in the cell) and vice versa, in the presence of 1-mM FAD. The resulting data were fitted to a one site binding model using the Origin software accompanying the ITC instrument.

### Analytical high-resolution size exclusion chromatography

The formation of complexes between MnmE and MnmG, as well as with tRNA, was followed using high-resolution analytical gel filtration experiments. Proteins and tRNA were mixed and, after 20-min incubation, 25 μl was injected on a KW803 gel filtration column (Shodex) coupled to an HPLC system (Waters). The separation was performed at a flow rate of 0.8 ml/min in a buffer consisting of 50-mM Hepes pH 7.5, 150-mM KCl, 5-mM MgCl_2_ and 5-mM BME. For the complex formation in the presence of GDP–AlFx, MnmE was incubated with 1-mM GDP–AlFx (1-mM GDP, 1-mM AlCl_3_ and 10-mM NaF) for 30 min and then mixed with MnmG.

### GTP hydrolysis assay

Hydrolysis of GTP by the MnmE(E282A)–MnmG complex was followed over time by separation of the nucleotides (GTP and GDP) using a C18 reversed phase column attached to an HPLC system (Waters). 66.7-μM *E. coli* MnmE(E282A)–MnmG complex was mixed with 100-μM GTP in a buffer consisting of 20-mM Hepes pH 7.5, 150-mM KCl, 5-mM MgCl_2_ and 2-mM DTT. At several time points, between 0 min and 24 h, 20-μl samples were taken and the reaction was stopped by flash freezing in liquid nitrogen. After boiling and centrifugation, the supernatant was loaded on a C18 reversed phase column (Jupiter, 25 cm x 4.6 mm) and the nucleotides were eluted using a buffer containing 100-mM KH_2_PO_4_ pH 6.4, 10-mM tetrabutyl ammonium bromide and 7.5% acetonitrile. Nucleotides were detected using absorbance at 254 nm. The GTP concentration in the sample was calculated from the peak area using a standard curve derived from known GTP concentrations. At each time point, also a second aliquot was taken, which was immediately analyzed using analytical high-resolution size exclusion chromatography (as described above), to assay complex formation between MnmE and MnmG.

## RESULTS

### Dimerization of the G domains of MnmE coincides with movements of the helical domains

**Figure 1.**
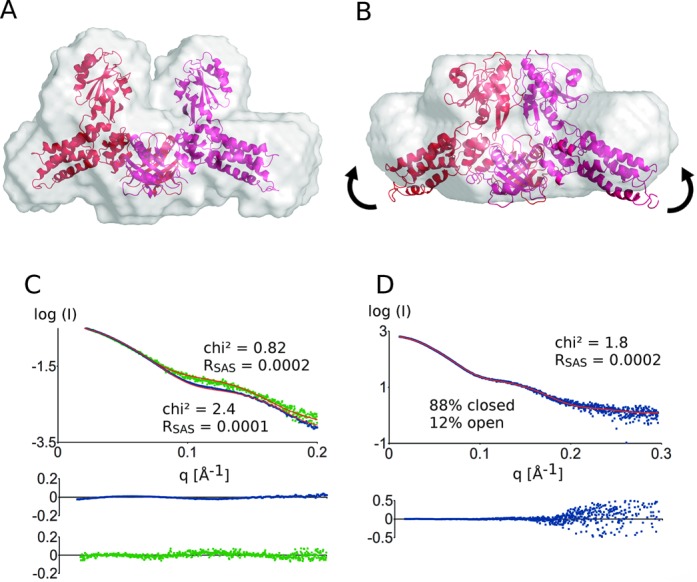
Model of *E. coli* MnmE in the (**A**) open and (**B**) closed states superposed on their *ab initio* shape as obtained by Dammif. The closure of the G domains probably also induces an ‘upward’ shift in the helical domains as indicated by the arrows. (**C**) Superposition of experimental and theoretical scatter curves using Crysol. The experimental scattering curves of the open (green) and the closed (blue) state of MnmE agree well with the theoretical scattering curves (red) obtained from the models. The residuals of the fitting are shown below the experimental curves in the corresponding color. (**D**) Fitting of the experimental scatter curve of MnmE bound to GppNHp to a mixture of MnmE in the open and closed states using OLIGOMER. A fraction of 88% of MnmE in the closed state and 12% of MnmE in the open state is obtained. The residuals of the fits are depicted below.

X-ray crystal structures of MnmE have until now been reported from *C. tepidum*, *Nostoc* and *Thermotoga maritima* (25). These structures show MnmE in the so called ‘open’ state, with the G domains of the homodimer facing—but not contacting—each other. However, crystal structures of the isolated G domains of *E. coli* MnmE bound to the transition state homologue GDP–AlFx showed that the G domains dimerize in the transition state (24). Supported by EPR studies and biochemical studies (25), this led to a model for MnmE action in which the protein cycles between a GDP-bound ‘open’ state and a GTP-bound ‘closed’ state. Unfortunately, until this date, no structures have been reported of a full-length MnmE in this closed state. In a first stage therefore, we set out to investigate the conformational behavior of *E. coli* MnmE in solution using SAXS measurements. Since currently no crystal structures of full-length *E. coli* MnmE are available, a homology model of this protein in the open state was generated starting from the existing crystal structure of *C. tepidum* MnmE (PDB code 3GEE) (25), with which it shares 36% sequence identity (Figure 1A).

SAXS data of the ‘open’ *E. coli* MnmE were collected in batch mode at the X33 beamline (Hamburg, Germany) in the absence of nucleotides. Analysis with Crysol and ScÅtter shows that the scattering curve of *E. coli* MnmE agrees very well with the homology model (chi^2^: 0.82; *R*_SAS_: 0.0002) validating the crystal structures of the open form of MnmE in solution (Figure 1C). Also the theoretical *R*_g_ (radius of gyration) of the model agrees well with the experimental *R*_g_ (*R*_gmodel_: 38.1 Å; *R*_gexp_: 38.6 Å ± 0.9 Å). Furthermore, the calculation of the molecular weight using either the SAXS data (MW_SAXS_) or SEC-MALS data (MW_MALS_) (Supplementary Figure S2 and Supplementary Table S2) supports that *E. coli* MnmE is a dimer in solution (MW_calc_: 103 kDa; MW_SAXS_: 104 kDa; MW_MALS_: 100 kDa). Subsequently, an *ab initio* molecular envelope was created from the experimental SAXS data using Dammif. This envelope overlays perfectly with the homology model supporting that the G domains of MnmE are separated in the open state (Figure 1A).

To gain insights into the structure of the closed state of *E. coli* MnmE, SAXS data were collected in the presence of 1-mM GDP–AlFx at the X33 beamline (Hamburg, Germany) in batch mode. Clearly distinct scattering curves are obtained for MnmE in the nucleotide-free (open) state and the GDP–AlFx-bound (closed) state (Figure 1C), indicative of considerable conformational changes in solution. Since no crystal structures were available for the closed state, we constructed a model, whereby the G domains were arranged as found in the crystal structure of the isolated G domains in the closed state (24), but leaving the other domains of MnmE unchanged (see Figure 1B). This model fits the experimental data reasonably well (chi^2^: 2.4; *R*_SAS_: 0.0001) and is clearly a better approximation of the structure in solution than the open form of MnmE, thus further supporting occurrence of the open–close transition of MnmE (Supplementary Figure S3). The *R*_g_ of the model agrees well with the experimental *R*_g_ (*R*_gmodel_: 37.0Å; *R*_gexp_: 37.2 Å ± 0.9 Å) and the calculation of the molecular weight supports that *E. coli* MnmE is also dimeric in the closed form (MW_calc_: 103 kDa; MW_SAXS_: 121 kDa; MW_MALS_: 97 kDa). Based on the experimental SAXS data an *ab initio* shape was calculated using Dammif (Figure 1B). This molecular shape confirms the dimerization of the G domains but suggests that an additional ‘upward’ movement of the helical domains of MnmE is taking place concomitant with the closing of the G domains (indicated by arrows in Figure 1B).

EPR studies have previously shown that, while MnmE mainly exists in the open state if GDP is bound and in the closed state if GDP–AlFx is bound, an equilibrium between both states exists in the presence of GppNHp, a ground-state analog of GTP (25). Correspondingly, an experimental SAXS curve of MnmE in the presence of 1 mM of GppNHp could neither be appropriately fitted to the model of the open state nor to the closed state. Therefore OLIGOMER was used to estimate the fractions of the open and closed forms of MnmE in the GppNHp-bound state (Figure 1D). This approach led to an optimal description of the SAXS data (chi^2^: 1.8; *R*_SAS_: 0.0002) using a ratio of 88% MnmE in the closed state and 12% in the open state. This fraction of MnmE in the closed form is higher than that found via EPR (about 30%), which might be due to the different conditions and temperatures that were used in both experiments.

### The solution structure of MnmG agrees well with the crystal structure

Currently crystal structures of MnmG have been reported from *E. coli*, *A. aeolicus* and *C. tepidum* (27–29). To confirm whether these crystal structures correspond to the protein structure in solution, SAXS data were collected. Unfortunately, slight aggregation of *E. coli* MnmG in the capillary hampered accurate interpretation of the scattering data. Therefore we turned to the homologous MnmG from *A. aeolicus* (51% sequence identity to *E. coli* MnmG) (PDB code 2ZXI) (28). Surprisingly, SEC-MALS data suggest that MnmG from *A. aeolicus* is mainly present as a monomer in solution, with only a minor dimer peak at the concentrations used [Supplementary Figure S2(F)]. However, since *E. coli* MnmG is clearly dimeric at all concentrations used [Supplementary Figure S2(D)], we focused our SAXS analysis on the dimer peak of *A. aeolicus* MnmG. Therefore, SAXS data were collected at the SWING beamline (Soleil, Paris) using an HPLC setup, in which purified MnmG is applied on a gel filtration column and the SAXS spectra were recorded after elution of the protein from the gel filtration column. The theoretical scattering curve of the *A. aeolicus* MnmG dimer (as observed in the crystal structures) shows a good fit to the experimental SAXS data (chi^2^: 1.9; *R*_SAS_: 0.03) (Supplementary Figure S4). The obtained *R*_g_ values (*R*_gmodel_: 40.2 Å; *R*_gexp_: 42.3 Å ± 1.5 Å) and the calculated molecular weights further support that *A. aeolicus* can also form a dimer in solution (MW_calc_: 150 kDa; MW_SAXS_: 168 kDa; MW_MALS_: 130 kDa). An *ab initio* molecular model was subsequently created from the data using Dammif. This envelope can be nicely superimposed on the crystal structure (Supplementary Figure S4).

### The MnmG dimer seems to bind a single tRNA molecule

**Figure 2.**
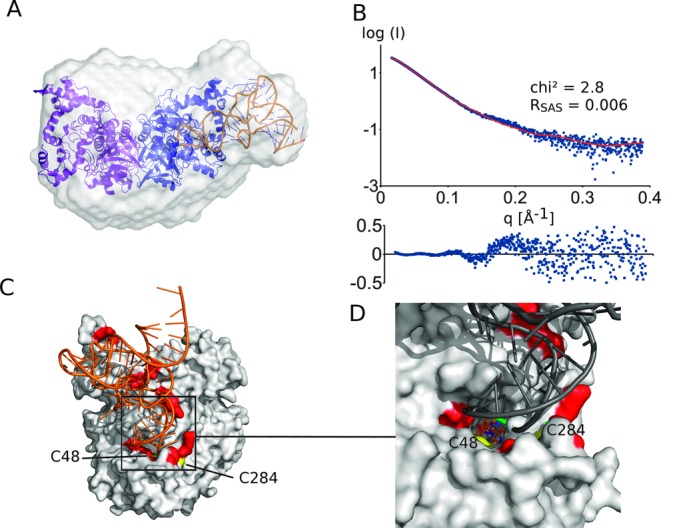
(**A**) Docking model of the *A. aeolicus* MnmG–tRNA complex superposed on the *ab initio* shape as obtained by Dammif. (**B**) The fit of the theoretical (red) to the experimental (blue) scattering curve supports the model. The residuals of the fits are also shown. (**C**) Details of the preferred model of the MnmG–tRNA interaction (compare to an alternative model in Supplementary Figure S5) and (**D**) a close-up view of the (FAD-binding) active site. This model is supported by the proximity of known tRNA-interacting residues (red) (28) and the catalytic cysteines (C48 and C284; yellow) and by the location of the FAD co-factor (shown as a stick model) with respect to the tRNA wobble nucleotide (green).

Within the context of the tRNA modification reaction, it has been shown that MnmG is mainly responsible for tRNA binding and can bind tRNA in the absence of MnmE (28). To prepare an MnmG–tRNA complex, we mixed *A. aeolicus* MnmG with a 1.2-molar excess of *A. aeolicus* tRNA^Lys^(UUU) and purified the complex via gel filtration. SAXS data of the fractions containing the MnmG–tRNA complex were afterward measured at the SWING beamline (Soleil, Paris) using an HPLC setup. In order to create a model of the MnmG–tRNA complex, first docking was performed using PatchDock. The MnmG–tRNA docking models were subsequently selected against the experimental SAXS data based on the chi^2^ value obtained by Crysol. This procedure led to two equally well scoring models that cannot be distinguished by SAXS (see Figure 2 and Supplementary Figure S5 for a comparison of the models). For both models, however, the data clearly suggest that only one subunit of MnmG is bound to a tRNA molecule, while the second subunit remains vacant. This is established by the good fit of the experimental data to a model with only one bound tRNA (chi^2^: 2.8; *R*_SAS_: 0.006), in comparison to a similar model with two bound tRNAs (chi^2^: 22; *R*_SAS_: 0.03) (Figure 2B and Supplementary Figure S6). Additionally, the *ab initio* shape created based on the experimental data with Dammif shows extra features on only one side of the MnmG molecule, in agreement with the presence of only one tRNA (Figure 2A). However, it might be possible that we did not saturate the binding sites of MnmG and that both tRNA-binding sites might still be occupied when using higher amounts of tRNA. In addition, we therefore also performed SEC-MALS experiments on the *A. aeolicus* tRNA and the *A. aeolicus* MnmG–tRNA complex. To this end, we mixed a 5-fold molar excess of tRNA with MnmG before injection on the size exclusion column. The molecular weight obtained for the *A. aeolicus* MnmG–tRNA complex is 151 kDa [see Supplementary Figure S2(H)], which is somewhat lower than the calculated molecular weight of one tRNA molecule binding to an MnmG dimer (174 kDa), but which corresponds to the sum of the experimental molecular weight from SEC-MALS of MnmG (130 kDa) and tRNA (25 kDa) [see Supplementary Figure S2(F) and (G)].

Despite the fact that we are unable to discriminate between the two best scoring MnmG–tRNA models (Supplementary Figure S5) solely based on the scattering data, we clearly prefer the model shown in Figure 2C based on mechanistic arguments. In this model, the anticodon stem-loop (ASL) of the tRNA, containing the substrate wobble uridine, is located in the FAD-binding pocket of MnmG close to the co-factor FAD and to the catalytic residues C48 and C248 (*A. aeolicus* numbering), of which the latter is situated at the end of a flexible loop [compare Supplementary Figure S5(C) and (D)] (28). This MnmG–tRNA model is further supported by a previous site-directed mutagenesis study that was used to map the tRNA-binding region of *A. aeolicus* MnmG (28). The tRNA-interacting residues that were identified in this way (e.g. Asn49, Arg97, Arg282, Lys290, Lys293, Arg439, Arg443, Tyr545 and Arg548; *A. aeolicus* numbering) coincide with the MnmG–tRNA interaction surface in our model (see Figure C2).

### MnmE and MnmG form an asymmetric, L-shaped complex

**Figure 3.**
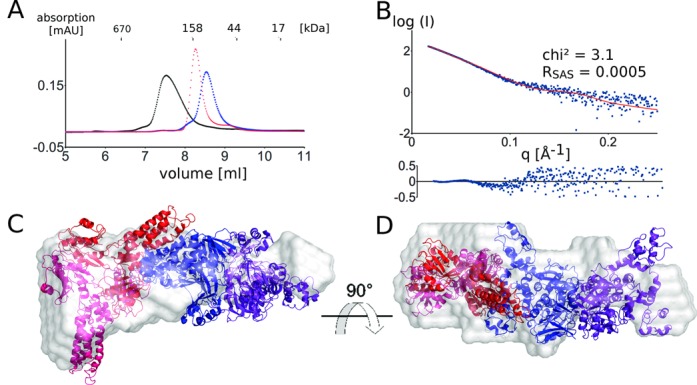
(**A**) Gel filtration experiment (KW-803, Shodex) showing the complex formation of the MnmE dimer (blue) and the MnmG dimer (red), forming the α2β2 MnmEG complex (black). Twenty five microliters of 100-μM solutions were injected on the column. (**B**) The theoretical scattering curve (red) of the MnmEG docking model agrees well with the experimental scattering curve (blue). The residuals of the fits are also shown. (**C**) and (**D**): Docking model of the α2β2 MnmEG complex superposed on the *ab initio* shape as obtained by Dammif (gray).

As already shown previously, MnmE and MnmG can form a tetrameric complex with α2β2 stoichiometry (i.e. 1-MnmE and 1-MnmG homodimer) in the GDP-bound state (23). Complex formation can be monitored by size exclusion chromatography, where a 1:1 mixture of MnmE and MnmG at 100 μM elutes at a smaller elution volume than the individual proteins, corresponding to a molecular weight of 260 kDa, close to the expected molecular weight (243 kDa) for the MnmE–MnmG α2β2 complex (Figure 3A). However, upon mixing of MnmE and MnmG at lower concentrations a gradual shift in the elution volume is observed from a volume corresponding to the molecular weight of the α2β2 complex to the elution volume corresponding to the individual proteins. This is typical for a low affinity complex with a very fast binding and dissociation rate. Moreover, at very high concentrations of MnmE and MnmG an even further decrease in elution volume, corresponding to a higher molecular weight complex, can be observed.

To get insights into the overall architecture of the α2β2 complex *E. coli* MnmE was mixed equimolar with *E. coli* MnmG and the resulting complex was purified via gel filtration. SAXS data were collected on this sample at the SWING beamline (Soleil, France) using an HPLC setup.

A model of the MnmEG complex was generated by docking of MnmG to MnmE. Hereto, we started from MnmE with its G domains in the closed form, since both EPR studies and stopped-flow fluorescence studies indicated that MnmG binding at least partially induces G domain dimerization in MnmE (23, 48). The resulting docking models were validated and ranked using experimental SAXS data, based on the chi^2^ value obtained by Crysol and on agreement with the *ab initio* shape as obtained by Dammif, and finally on visual inspection of the models in terms of functionality (proximity of active sites, vacancy of the tRNA-binding site on MnmG—see Supplementary material and methods for a detailed description). This led to the model shown in Figure 3C and D. Considering the size of the complex and the fact that we cannot exclude the occurrence of conformational changes in both proteins upon complex formation, a good fit of the theoretical and experimental scatter curves is obtained (chi^2^: 3.1; *R*_SAS_ = 0.0005) (Figure 3B). The 1:1 stoichiometry of the model is supported by the radius of gyration (*R*_gmodel_: 51.2 Å; *R*_gexp_: 52.3 Å ± 1.4 Å) and the molecular weight (MW_calc_: 243 kDa; MW_SAXS_: 207 kDa) and is also further confirmed by the molecular weight obtained by SEC-MALS measurements (MW_MALS_: 213 kDa; Supplementary Figure S2). The fact that the experimental molecular weights are somewhat smaller than the calculated molecular weight probably indicates that at the concentration used, the complex formation is not completely saturated.

In this model, one MnmE dimer is binding via the N-terminal domain and the helical domain of one subunit to the C-terminal domain (mainly helix α11 and α19) of one subunit of the MnmG dimer in a non-symmetric manner (Figure 3C). Interestingly, this model leaves one protein-binding site vacant on MnmE as well as one on MnmG, allowing for further oligomerization. The fact that such oligomerization is not observed below 200 μM (in the absence of nucleotides, see further) indicates a form of negative cooperativity between the protomers of both MnmE and MnmG. A similar negative cooperativity was also observed between the subunits of MnmG with regard to tRNA binding. However, contrary to the previously proposed models for the MnmE–MnmG interaction (27), in our current model the 5,10-methylenetetrahydrofolate-binding site of MnmE and the FAD-binding site of MnmG are oriented toward each other. Such an arrangement would be required to be conform with the proposed mechanism of tRNA modification by MnmEG (6, 9), which assumes an intensive collaboration between both active sites. Finally it should be noted that the MnmEG interaction model that was previously proposed (27) does clearly not fit with the experimental SAXS data (chi^2^: 30.3; *R*_SAS_ = 0.01) (Supplementary Figure S7).

**Figure 4.**
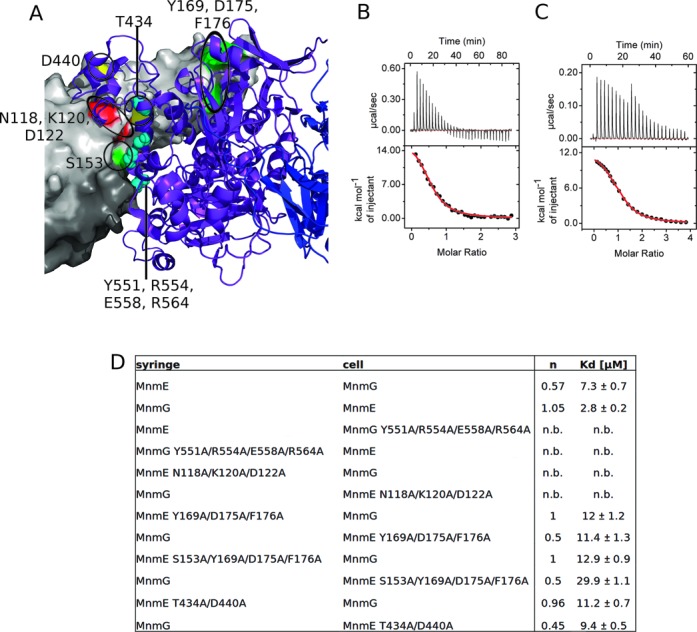
Validation of the MnmEG complex interface using ITC. (**A**) Residues that showed a decrease in affinity upon mutation to alanine are indicated as followed - red: MnmE (N118A, K120A and D122A); green: MnmE (Y169A, D175A and F176A) with additional S153A; yellow: MnmE (T434A and D440A); cyan: MnmG (Y551A, R554A, E558A and R564A). MnmE is shown with its surface in light and dark gray and MnmG is shown as a cartoon model in purple and blue. ITC data of the titration of (**B**) MnmE wt to MnmG wt and (**C**) MnmG wt to MnmE wt, showing the dependence of the binding stoichiometry on the direction of titration. (**D**) Summary of binding stoichiometry (*n*) and apparent *K*_d_ values of MnmE/MnmG variants as obtained by ITC. n.b. = no binding detected.

In order to validate our model we used ITC measurements. Wild-type MnmE binds MnmG with an apparent dissociation constant (*K*_D_) of 3 μM and 7 μM, depending on the direction of the titration (Figure 4). To our surprise, we observed that the stoichiometry of the complex consistently depends on the direction of the titration. Adding MnmG (in the syringe) to MnmE (in the cell) leads to a stoichiometry of 1, which is consistent with a 1:1 complex (23). However adding MnmE to MnmG leads to a stoichiometry of 0.5. This observation was repeatable with different batches of protein, but we do not have a fully adequate explanation so far. However, such a phenomenon has been observed before in the case of systems capable of forming long alternating chains of homodimers, like ccdA/ccdB (49). In that specific case, it was indicative of kinetic limitations that prevent reaching equilibrium in at least one direction of the titration. Here, a full interpretation only became possible after high resolution structures of the ccdA/ccdB protein complexes were solved.

Subsequently we measured the effect on the binding affinity of point mutations of conserved residues in the proposed binding interface (Figure 4 and Supplementary Figure S8). It should be stressed here that the SAXS-based modeling only provides low-resolution shapes of the complexes between MnmE and MnmG and thus any detailed information concerning individual atomic interactions is missing. As pointed out before we can also not exclude the occurrence of small conformational changes in the individual proteins upon complex formation. To more easily pinpoint the interaction surfaces by mutagenesis we therefore chose to construct multiple mutants, hence covering a whole patch on the proposed interaction surface. To exclude the possibility that these mutations have an influence on the overall structure and stability of the proteins, rather than disrupting the actual binding interface, we performed thermal unfolding experiments (Supplementary Figure S9). All mutants show a melting temperature that is comparable to the corresponding wild-type proteins, in the range of 47°C. As the ITC experiments were performed at 5°C, these data support that any effect of the mutations on binding is not due to a destabilizing effect on the proteins. The strongest decrease in (apparent) affinity is observed for the mutants MnmE (N118A, K120A and D122A) and MnmG (Y551A, R554A, E558A and R564A) that completely abolish binding at the concentrations used in our ITC experiment. Both patches of residues are located in the middle of the proposed MnmEG interface and are in very close proximity to each other. Mutations that are located at the periphery of the proposed interaction surface clearly decrease affinity to a lesser extend. The mutant MnmE (Y169A, D175A and F176A), located near the outer end of the helical domain, shows a 2–4-fold decrease in affinity (depending of the direction of the titration). An additional mutation of Ser153 to alanine, located at the edge of the N-terminal and helical domain, decreases the (apparent) affinity even further (up to 10-fold compared to wt MnmEG). Also the mutant MnmE (T434A and D440A), located between the N-terminal domain and the helical domain, decreased the (apparent) affinity toward MnmG (2–3-fold). Finally, in our model the C-terminal end of MnmG (amino acids 550–629) is also involved in the interaction with MnmE. It has previously been shown that deletion of this part of MnmG severely affects binding of MnmG to MnmE (29). Altogether these mutagenesis data support the MnmEG model proposed here. It is noteworthy that all mutants of MnmE that showed an effect on the binding affinity also reversed the stoichiometry of titration compared to wild-type MnmEG. The underlying mechanism for this observation remains thus far obscure.

### GDP–AlFx binding induces formation of an α4β2 complex

**Figure 5.**
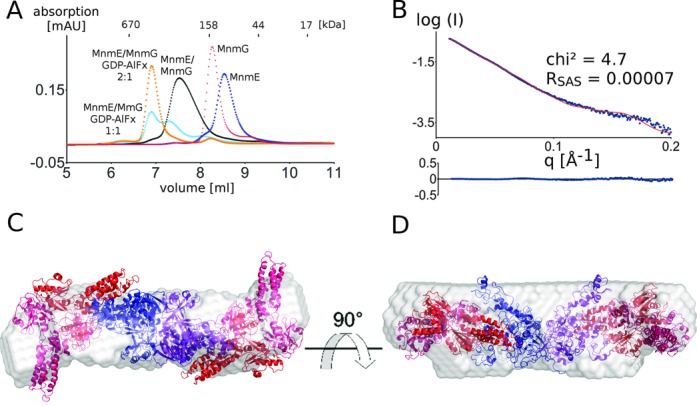
(**A**) MnmE and MnmG form a α4β2 complex in the presence of GDP–AlFx. A single species on gel filtration is only obtained if MnmE is used in 2:1 excess over MnmG (orange) while a mixture of species is obtained in a 1:1 stoichiometry mixing (cyan). Twenty-five microliters of 100-μM solutions were injected on the column. (**B**) The theoretical scattering curve of the α4β2 model (red) agrees with the experimental scattering curve (blue). The residuals of the fits are also shown. (**C**) and (**D**): Model of the α4β2 MnmEG complex superposed on the *ab initio* shape as obtained by Dammif (gray).

To further assess the influence of the nucleotide state (GTP versus GDP) of MnmE on its binding to MnmG, the MnmEG complex was analyzed in the presence of GDP–AlFx. High-resolution size exclusion chromatography suggests the formation of an even larger MnmEG complex when MnmE is bound to GDP–AlFx, even at very low protein concentrations (5 μM) (Figure 5A). The apparent molecular weight of this complex according to gel filtration is 520 kDa, compared to 260 kDa determined for the α2β2 complex. Interestingly, a single peak for this larger complex on gel filtration could only be observed if MnmE was used in 2-fold excess over MnmG. A 1:1 mixture of MnmE–GDP–AlFx and MnmG led to the formation of a peak for this high molecular weight complex in addition to several other peaks at lower molecular weight.

To gain insights into the overall architecture and shape of this larger complex, SAXS data were collected on the MnmE–GDP–AlFx–MnmG complex. Therefore MnmE–GDP–AlFx was mixed in 2-fold molar excess with MnmG and SAXS data were collected at the SWING beamline (Soleil, France) in an HPLC setup. The molecular weight as determined by SAXS was 338 kDa, which is in agreement with the molecular weight estimate from SEC-MALS (326 kDa) and corresponds to a 2:1 (two MnmE dimers and one MnmG dimer) complex (MW_calc_: 346 kDa). This molecular weight estimate is smaller than the estimate obtained from gel filtration. However, the molecular weight estimations of SAXS and MALS are independent of the shape of the molecule, whereas in gel filtration the extended nature of the α4β2 complex (see following) probably leads to the aberrant elution. We created a tentative model with 2:1 (MnmE:MnmG) stoichiometry by placing a second MnmE dimer on the vacant binding site of MnmG in our α2β2 model (Figure 5C and D). The theoretical scattering curve of the resulting model is in very good agreement with the experimental scattering curve (chi^2^: 4.7; *R*_SAS_: 0.0007), especially considering that possible smaller conformational changes upon complex formation in MnmE and MnmG are not taken into account (Figure 5B). The model is further supported by the *R*_g_ obtained from the model compared to the experimental *R*_g_ (*R*_gmodel_: 67.2 Å; *R*_gexp_: 67.4 Å ± 1.3 Å). Finally, the *ab initio* molecular envelope obtained by Dammif agrees very well with the α4β2 model and shows the elongated nature of this complex (Figure 5C and D). As a control and to rule out that this larger complex is formed by the addition of two MnmG dimers to the vacant binding sites of one MnmE, we created a model with a 1:2 stoichiometry (MnmE:MnmG). The fitting statistics clearly indicate that this model is incorrect (chi^2^: 303; *R*_SAS_: 0.12; *R*_gmodel_: 57.9 Å; *R*_gexp_: 67.4 Å ± 1.3 Å) and the model is also not in agreement with the *ab initio* envelope obtained by Dammif (not shown).

Attempts to obtain a homogeneous sample of the MnmEG–tRNA complex did not succeed. Therefore, to get insights into this MnmEG–tRNA complex we generated a tentative model based on the previous models (Supplementary Figure S10). Since two tRNA-binding interfaces in the α4β2 complex are available, we suspect that this complex will bind two tRNA molecules. Therefore we superposed the MnmG–tRNA model (Figure 2A) onto the model of the MnmE–GDP–AlFx–MnmG complex (Figure 5C). This tentative model would create some overlap between tRNA and MnmE. However, these sterical clashes could be relieved by the conformational changes in the α-helical domains of MnmE that are anticipated upon GTP binding (see Figure 1B). This low-resolution architecture places the wobble position of the tRNA in between the 5,10-MTHF- and FAD-binding pockets of MnmE and MnmG, as would be required for the modification reaction to occur. The proposed movement of the α-helical domain of MnmE upon GTP binding might bring the 5,10-MTHF in even closer contact to the wobble uridine of tRNA. Interestingly it should be noted that the MTHF closest to the wobble uridine of tRNA is not coming from the MnmE protomer interacting via its α-helical domain with MnmG, but is coming from the second MnmE protomer.

### Nb_MnmG_1 binds MnmG and disrupts the MnmE–MnmG complex

A camelid single domain antibody fragment (Nanobody), which we named Nb_MnmG_1, was identified to bind *E. coli* MnmG. Interestingly, gel filtration experiments show that Nb_MnmG_1 can disrupt the MnmEG complex, both in the α2β2 and α4β2 forms, when added in excess [Supplementary Figure S11(A)]. This finding suggests that Nb_MnmG_1 and MnmE are competing for the same binding surface of MnmG and can hence be used to further validate our MnmEG model.

An ensemble of MnmG–Nb_MnmG_1 models was created by docking of an Nb_MnmG_1 homology model to MnmG and the resulting models were selected based on the experimental SAXS data of the MnmG–Nb_MnmG_1 complex. The resulting best model, with Nb_MnmG_1 bound to MnmG in a 1:1 stoichiometry, is in good agreement with the experimental scattering curve (chi^2^: 3.6; *R*_SAS_: 0.0004) and with the *ab initio* model created with Dammif [Supplementary Figure S11(B) and (C)]. This is in agreement with the obtained *R*_gs_ (*R*_gmodel_: 42.6 Å; *R*_gexp_: 43.3 Å ± 0.4 Å) and the calculation of the molecular weight from the SAXS data, as well as from the SEC-MALS data (MW_calc_: 173 kDa; Mw_SAXS_: 184 kDa; MW_MALS_: 170 kDa). In this model, Nb_MnmG_1 is binding to the C-terminal helical domain of MnmG, on a position that indeed partially overlaps with the binding surface of MnmE in our MnmEG model. This finding hence further confirms the validity of our proposed MnmEG interaction model.

### Conversion of the α2β2 and α4β2 complexes is linked to GTP binding and hydrolysis

In the previous experiments, we have observed that MnmE and MnmG mainly form an α4β2 complex upon binding of GDP–AlFx, while an α2β2 complex is formed upon binding of GDP at the the concentrations used in the gel filtration experiments. On the other hand, under physiological conditions MnmE will actively hydrolyze the bound GTP and it has been previously described that this GTP hydrolysis is required for tRNA modification (25). We were therefore interested to see whether the conversion of the α4β2 complex into the α2β2 complex is linked to GTP hydrolysis.

**Figure 6.**
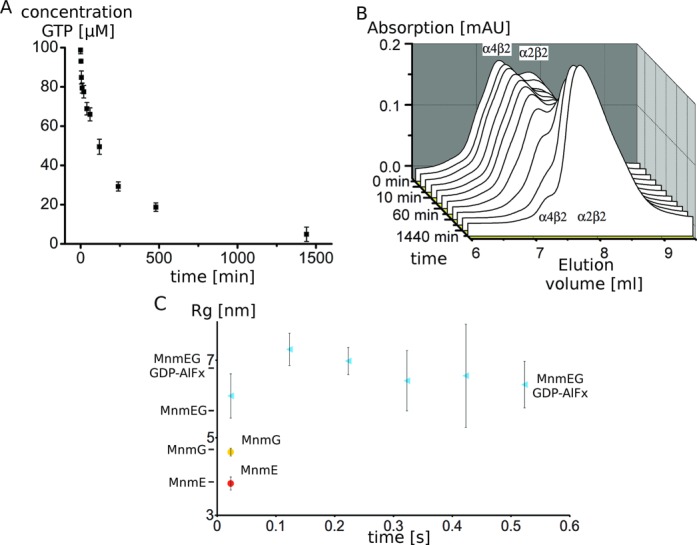
(**A**) GTP hydrolysis by the MnmE(E282A)–MnmG complex shows a similar time dependency to (**B**) the concomitant conversion of the MnmE(E282A)–MnmG complex from the α4β2 to the α2β2 form during GTP hydrolysis. (**C**) Analysis of MnmE–MnmG complex formation using stopped-flow SAXS. The evolution of the *R*_g_ in function of time upon mixing of MnmE and MnmG in the presence of GDP–AlFx (cyan) is shown and compared with the *R*_g_ of MnmE (red) and MnmG (yellow) upon mixing with buffer. The *R*_g_ values of the α2β2 and α4β2 complexes derived from static SAXS measurements are indicated at the side of the Y-axis. These data show that the formation of the α4β2 complex occurs very fast, within the dead time of the measurement (<20 ms). Error bars show the standard deviation of three measurements.

Since GTP hydrolysis by wild-type *E. coli* MnmE occurs on time scales that are too fast to follow via gel filtration experiments, a slow hydrolyzing mutant MnmE (E282A) was used (24). The MnmE(E282A)–MnmG complex was mixed with a small excess of GTP and both the GTP hydrolysis and the oligomeric state of the protein complex were followed and compared over time (Figure 6A and B). At time point zero, the MnmE(E282A)–MnmG complex is mainly GTP bound and correspondingly, mainly the α4β2 complex is observed (although some α2β2 complex is observed due to the equimolar mixing of MnmE with MnmG) (Figure 6B). After 24 h all the complex is converted to the α2β2 form and at intermediate time points a gradual shift from the large to the smaller complex is observed. This conversion in oligomeric state nearly perfectly corresponds to the hydrolysis of GTP by MnmE (E282A), which shows that all GTP is hydrolyzed after 24 h with a 50% GTP conversion after roughly 2 h (Figure 6A).

This clearly indicates that GTP hydrolysis and complex disassembly are directly linked. However, the latter also implies that for the wild-type MnmEG complex, formation of the α4β2 complex from the α2β2 complex will need to compete with the disassembly due to GTP hydrolysis [*k*_cat_ = 0.16 s^−1^ (23)]. In order to be physiologically relevant, the α4β2 complex must hence form in a time scale faster than or comparable to GTP hydrolysis. To gain insights into how fast the complexes assemble, a stopped-flow setup directly coupled to SAXS beamline P12 (EMBL, Hamburg) was used. In this setup, MnmE and MnmG were rapidly mixed in the presence of GDP–AlFx and SAXS spectra were recorded at different time points after mixing (50-ms exposures). As control, mixing of either MnmE or MnmG with buffer resulted in time invariable *R*_gs_ of 3.82 nm and 4.63 nm, respectively, very close to the values obtained in a static HPLC setup (3.87 nm and 4.36 nm, respectively). Mixing of MnmE with MnmG in the presence of GDP–AlFx led to the formation of a large complex with an *R*_g_ fluctuating around 6.5 nm, in agreement with the formation of the α4β2 complex (Figure 6C). It must, however, be noted here that under the high protein concentration that is required in this experiment (due to the very low exposure times), even in the absence of GDP–AlFx the equilibrium of the MnmEG complex seems to be shifted to the α4β2 complex (data not shown). In either case, formation of the α4β2 complex upon mixing of MnmE and MnmG occurs very fast and within the dead time (<20 ms) of our measurements. This thus strongly suggests that the rate of formation of this complex can compete with GTP hydrolysis and concomitant disassembly of the complex and that the α4β2 complex would hence be populated and relevant under active GTP turnover conditions.

## DISCUSSION

The survival of any organism depends on the efficient and correct translation of mRNA into protein. In this process, the proper recognition of the codon (especially in split codon boxes) is immediately linked to the modification at the wobble position of tRNA. Hence, defects in wobble modification are often correlated with diseases in humans (18).

MnmE and MnmG are responsible for the cmnm^5^U34 wobble modification and act in a concerted manner (23). Although crystal structures of the single proteins were being unraveled starting from 2005, it has proven to be very difficult to understand how they collaborate in the tRNA modification reaction. It was known that the proteins could form an α2β2 complex (23), but no structures of the MnmEG complex nor of complexes with the tRNA substrates have been reported to date.

**Figure 7.**
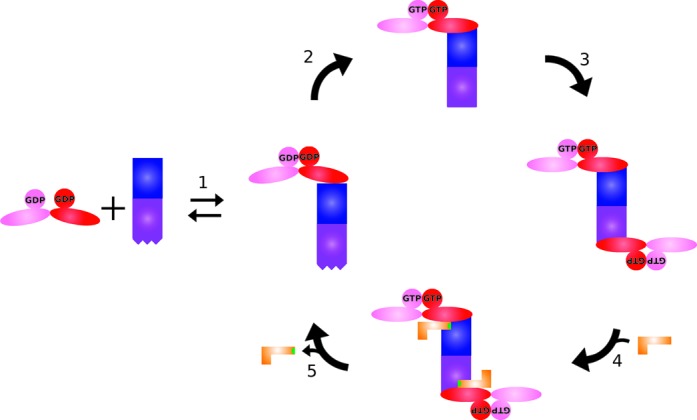
Model of MnmEG complex formation during the tRNA modification cycle (MnmE is colored red and pink; MnmG blue and purple). **(1) MnmE is binding to MnmG in a non-symmetric manner with initial closure of the G domains. (2) Upon GTP binding the G domains of MnmE undergo further closing, with a concomitant movement of the helical domain, leading to a structural change in MnmG, (3) promoting the binding of a second MnmE dimer on the other MnmG subunit. (4) In the following step two tRNA molecules can bind mainly via interactions with MnmG and (5) upon GTP hydrolysis the tRNA becomes modified (indicated in green), the complex dissembles to its α2β2 form and the tRNA leaves the complex.**

Here, we used solution SAXS studies to reveal the interaction and interplay between MnmE and MnmG at low resolution. Based on our studies, we propose a model (Figure 7) for the different steps that occur during the tRNA modification reaction by MnmEG. Based on biochemical and EPR studies (24,25), it was previously suggested that MnmE undergoes a transition from an ‘open’ (GDP-bound) to a ‘closed’ (GTP-bound) conformation. Our SAXS analysis corroborates this and clearly distinguishes between these two conformations (Figure 1). We moreover observe that this transition does not only include the closure of the G domains but is also coupled to a movement of the helical domains toward the G domains. Our SAXS data further clearly support that in a first step of the tRNA modification cycle (Figure 7, step 1) MnmE binds to MnmG in an asymmetric manner, leaving one subunit vacant on each MnmE and MnmG dimer (Figure 3). This observation suggests a form of negative cooperativity between the subunits of MnmE and MnmG, the nature of which is at this point not obvious from the available crystal structures. However, our model for the MnmEG α2β2 complex is further supported by mutagenesis studies and by the disruption of the complex by the binding of Nb_MnmG_1, which shares its MnmG interaction interface with MnmE. Moreover, the same binding asymmetry is observed in the binding of tRNA to MnmG, where only one tRNA molecule seems to bind to the MnmG homodimer (Figure 2). When GTP binds to MnmE in the α2β2 complex, the conformational changes in MnmE seem to induce allosteric changes on MnmG, since it promotes the binding of a second MnmE dimer on the opposite side of MnmG, resulting in an α4β2 complex even at low protein concentrations (Figure 7, step 3). The formation of this larger complex in the GTP-bound state is supported by high-resolution gel filtration experiments, SEC-MALS and SAXS measurements (Figure 5). In the next step, each MnmG monomer in the α4β2 complex can bind one tRNA leading to an α4β2γ2 complex (Figure 7, step 4 and Supplementary Figure S10). During (or prior to) GTP hydrolysis the tRNA will be modified at the wobble position and concomitant with GTP hydrolysis the large complex will dissociate again to an α2β2 form (Figure 7, step 5). This link between GTP hydrolysis and the conversion from the α4β2 to the α2β2 complex is clearly shown in Figure 6. Considering the relatively low affinity between MnmE and MnmG (*K*_D_ ≈ 3–7 μM in the presence of FAD) we cannot exclude that the α2β2 complex dissociates further into the MnmE and MnmG homodimers. Conversely, considering that (i) the cellular GTP concentration [1 mM–1.6 mM; (50,51)] is about 10 times higher than the GDP concentration, (ii) the GTP binding and MnmEG complex formation is fast and (iii) the dissociation of the G domains is rather slow (26), we can presume that MnmE will be for a considerable fraction in the ‘closed’ state in the cell and that hence the MnmEG complex will reside for a significant fraction in the α4β2 form.

To unravel the physiological relevance and advantages of these complexities in the MnmEG-catalyzed tRNA modification cycle, further detailed studies as well as high-resolution crystal structures of these protein and protein–tRNA complexes are required. This will also help to further understand the detailed molecular mechanisms behind the GTP-induced conformational changes promoting the oligomerization.

It should be noted in this regard that MnmE is not the only G protein that undergoes an oligomerization step during its functional cycle. Dynamin and DNM1L (dynamin 1-like protein) are, like MnmE, G proteins belonging to the class of G proteins activated by nucleotide-dependent dimerization (GADs) (20). Both proteins oligomerize via their α-helical stalks to form a large helical arrangement surrounding the ‘neck’ of a membrane vesicle, and dimerization of the G domains subsequently links neighboring turns of the dynamin helix (52–55). In contrast to these latter two G proteins, our data suggest that MnmE does not oligomerize in itself via its α-helical bundle domains. Conversely, two MnmE molecules use their α-helical bundles to bind one MnmG molecule as a connector. In the case of dynamin, the oligomerization is necessary for its function in membrane scission during endocytosis (52). The exact role of the oligomerization of the MnmEG complex during the tRNA modification cycle remains to be explored.

## SUPPLEMENTARY DATA

Supplementary Data are available at NAR Online.

## ACKNOWLEDGMENTS

We would like to thank the staff at the beamline ID14-3 of the ESRF in France, at the beamline SWING of Soleil in France and at the beamline P12 and X33 of Desy in Germany for assistance during data collection. We would like to thank Prof. Savvas N. Savvides (UGent) for access to the SEC-MALS apparatus and Yehudi Block for assistance.

## Supplementary Material

SUPPLEMENTARY DATA
