# Supplementary material for: SAXS analysis of the tRNA-modifying enzyme complex MnmE/MnmG reveals a novel interaction mode and GTP-induced oligomerization
Source: Nucleic Acids Res. 2014 Mar 14;42(9):5978–92. doi: 10.1093/nar/gku213 (PMC4027165; doi:10.1093/nar/gku213)
Supplement: SUPPLEMENTARY DATA [file supp_42_9_5978__index.html]

SAXS analysis of the tRNA-modifying enzyme complex MnmE/MnmG reveals a novel interaction mode and GTP-induced oligomerization — SAXS analysis of the tRNA-modifying enzyme complex MnmE/MnmG reveals a novel interaction mode and GTP-induced oligomerization — SAXS analysis of the tRNA-modifying enzyme complex MnmE/MnmG reveals a novel interaction mode and GTP-induced oligomerization — SUPPLEMENTARY DATA 

# SAXS analysis of the tRNA-modifying enzyme complex MnmE/MnmG reveals a novel interaction mode and GTP-induced oligomerization

## SUPPLEMENTARY DATA

**Files in this Data Supplement:**

- SUPPLEMENTARY DATA
