# Supplementary material for: SAXS analysis of the tRNA-modifying enzyme complex MnmE/MnmG reveals a novel interaction mode and GTP-induced oligomerization
Source: Nucleic Acids Res. 2014 Mar 14;42(9):5978–92. doi: 10.1093/nar/gku213 (PMC4027165; doi:10.1093/nar/gku213)
Supplement: SUPPLEMENTARY DATA [file supp_gku213_nar-03279-v-2013-File009.pdf]

Supplementary material:

SAXS analysis of the tRNA-modifying enzyme complex MnmE/MnmG reveals a novel interaction mode and GTP-induced oligomerization

Marcus Fislage, Elke Brosens, Egon Deyaert, Alessandro Spilotros, Els Pardon, Remy Loris, Jan Steyaert, Abel Garcia-Pino and Wim Versées

Supplementary material and methods:

Docking of MnmE to MnmG and selection of the models using SAXS data

Docking of MnmE to MnmG was performed using PatchDock (*Schneidman-Duhovny, D., Inbar, Y., Nussinov, R. and Wolfson, H.J. (2005) PatchDock and SymmDock: servers for rigid and symmetric docking. Nucleic Acids Res.,* ***33****, W363–367*). Hereby, the two partners are docked based on shape matching algorithms and the docking solutions are ranked according to a geometric shape complementarity score. After the docking, the program applies a clustering algorithm, which clusters models that are similar within an rmsd of 4 Å into one docking solution. This procedure resulted in the generation of 413 docking models for the MnmE-MnmG $\alpha 2\beta 2$ complex. Those models were fit against the experimental SAXS data of the MnmEG complex using Crysol, resulting in chi² values ranging from 4 to 60 (Figure S12 A&C). Figure S13 shows the top20 docking solutions ordered according to the chi² values. Afterwards the docking solutions were visually evaluated based on the overall fit of the model with the *ab initio* Dammif envelope, obtained from the SAXS data. Starting from the 13th-ranked model onward, one can observe significant deviation from the *ab initio* envelope, making the docking solution unlikely to be correct. Moreover, we initially de-prioritized docking models where MnmG interacts with the G domain of MnmE, since it has been clearly shown that the G domains of MnmE do not bind to MnmG, while the N-terminal domains together with the α-helical domains do (*Meyer S, Scrima A, Versées W, Wittinghofer A., J Mol Biol. 2008 Jul 11;380(3):532-47*). This led to the de-prioritization of several of the top20 models (Rank 1,2,4,5,8-11,14-17,19,20). We furthermore looked for conservation scores of residues in the proposed interfaces, leading to the rejection of further models. Moreover, models in which the tRNA binding site of MnmG is completely blocked by its interaction with MnmE were initially rejected. From the models that fit our criteria, the model ranked

6th after Crysol was the best scoring docking solution. Moreover, this solution scored 8th in the docking ranking, showing that this is a plausible interaction interface for the MnmE-MnmG complex.

Based on our selection we performed mutagenesis on MnmE and MnmG in the proposed binding interface and tested the influence of the mutations on the apparent binding affinity using ITC. The ITC data support the interaction mode of our preferred model (Figure 4). Finally, we re-evaluated the docking solutions based on the knowledge that MnmE and MnmG form an $\alpha 4 \beta 2$ complex if MnmE is bound to GDP-AlFx. Therefore we created for all top20 ranked solutions after Crysol an $\alpha 4 \beta 2$ model, by placing symmetrically a second MnmE on the vacant MnmG subunit. Those models were fit against the SAXS data of the $\alpha 4 \beta 2$ MnmEG-GDP-AlFx complex and only our preferred model results in an acceptable fit (chi^2: 4.7), while all the other models resulted in chi^2 values bigger than 32 (Figure S12 B&C). This further supports that our preferred and selected model represents the correct interaction mode of MnmE and MnmG.

CD, DLS and SEC-MALS

Dynamic light scattering (DLS) data of MnmE and the MnmEG complex were collected at 20°C in 10 mm cylindrical cuvettes at an angle of 90°C employing an ALV-CSG-3 static and dynamic light scattering device using a 22 mW He-Ne laser with a wavelength of 623.8 nm in a buffer consisting of 50 mM Hepes pH 7.5, 150 mM KCl, 5 mM $MgCl_2$, 1 mM DTT.

Thermal unfolding curves of MnmE and MnmG were recorded between 20°C and 80°C using the JASCO J-715 circular dichroism (CD) spectrometer. Therefore, at 1°C temperature intervals the CD signal at 224 nm (MnmE) or at 217 nm (MnmG) was recorded and plotted in function of the temperature. The melting temperature of each variant was then derived from the maximum of the 1st derivative of this curve.

Size exclusion chromatography multi-angle light scattering (SEC-MALS) experiments were performed using the Wyatt MALS system coupled to an Agilent 1250 Infinity pumping system and a Superdex 200 Increase 10/300 column (GE Lifesciences) at a flow rate of 0.5 ml/min in a buffer consisting of 50 mM Hepes pH 7.5, 150 mM KCl, 5 mM $MgCl_2$ and 1 mM DTT. Molecular weights (MW_{MALS}) were estimated using a dn/dc of 0.185 (Figure S2).

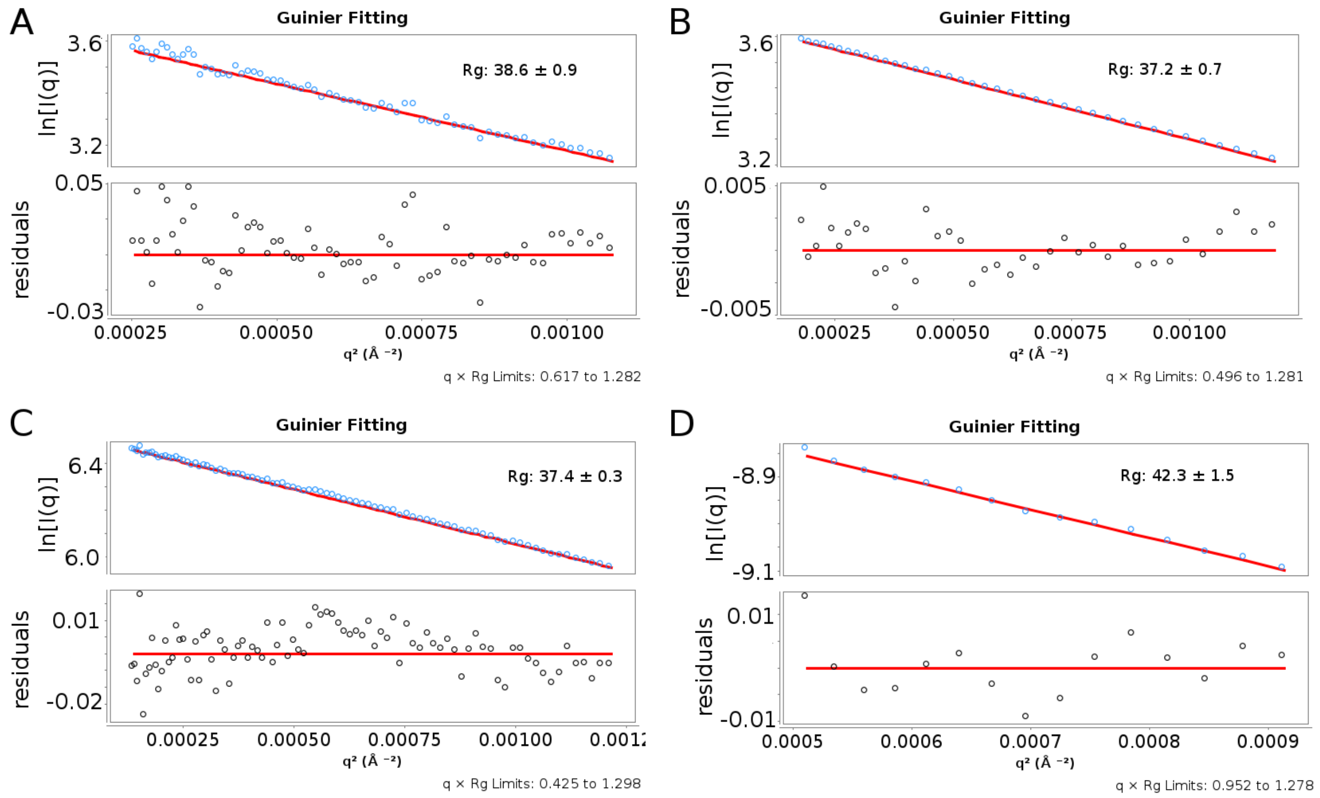

Figure S1: Guinier plots of (A) *E.c.* MnmE, (B) *E.c.* MnmE GDP-AlFx, (C) *E.c.* MnmE GppNHp, (D) *A.a.* MnmG

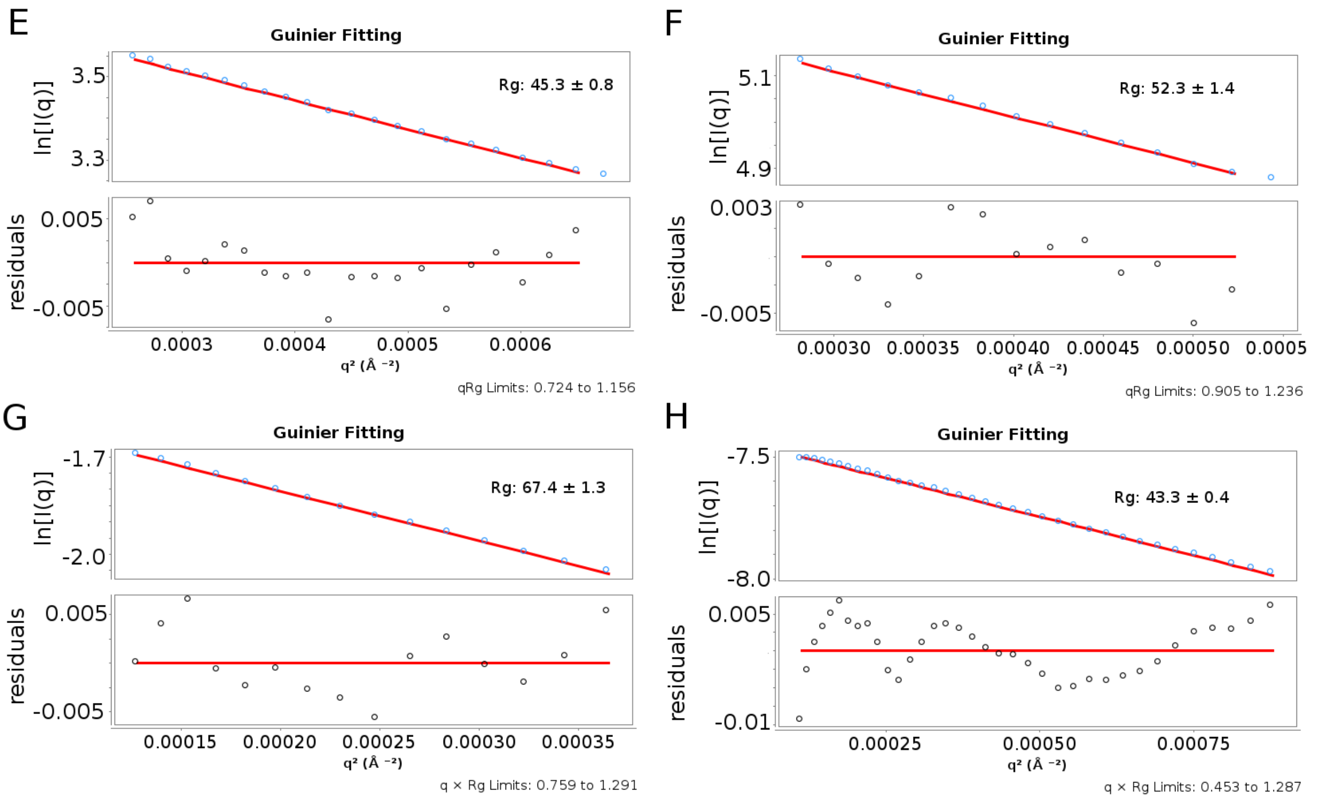

Figure S1 (continued): Guinier plots of (E) *A.a.* MnnmG + tRNA, (F) *E.c.* MnmEG, (G) *E.c.* MnmEG-GDP-AlFx, (H) *E.c.* MnmG + Nb_MnmG_1

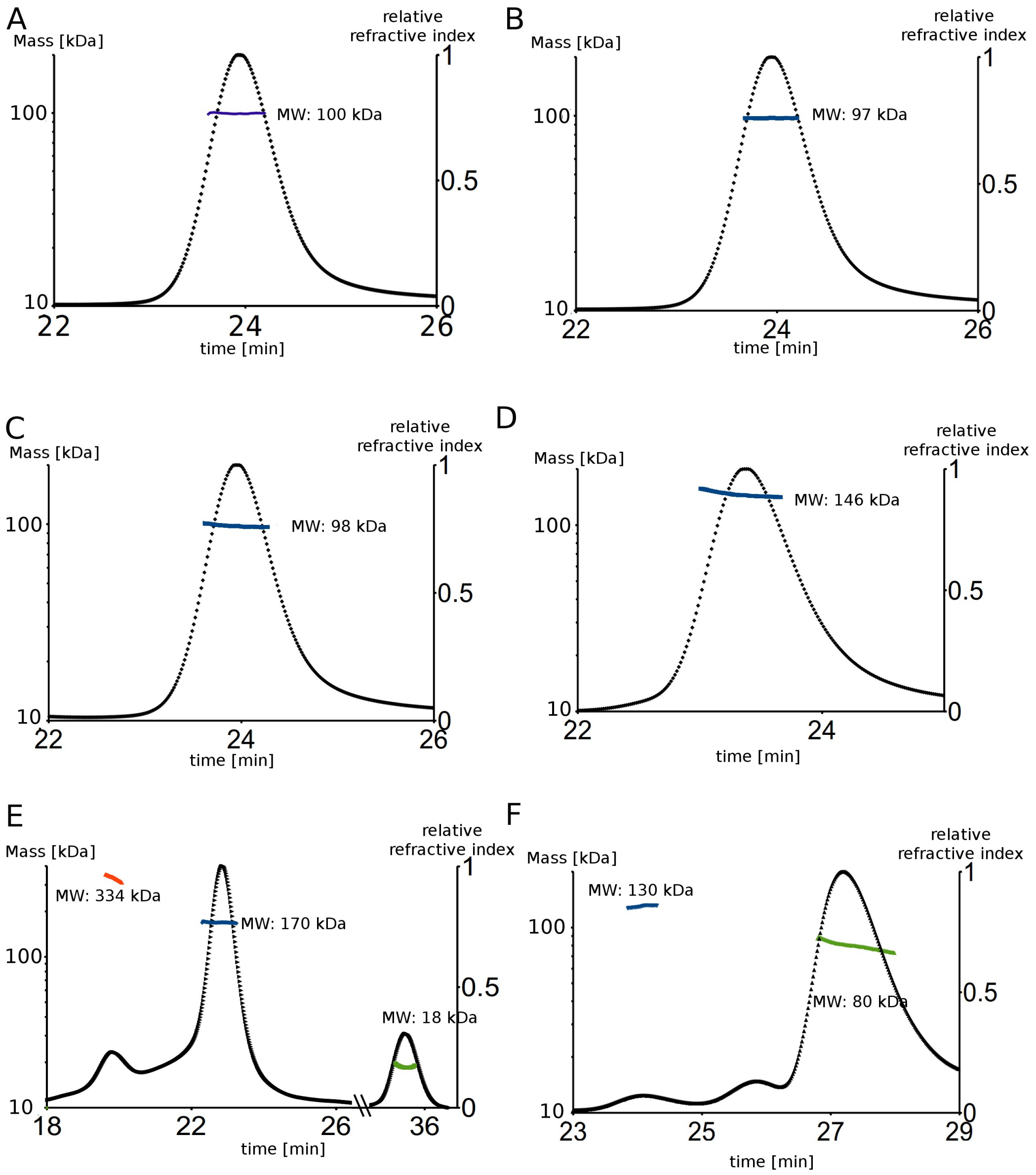

Figure S2: SEC-MALS data of (A) *E.c.* MnmE open, (B) *E.c.* MnmE closed, (C) *E.c.* MnmE GppNHp, (D) *E.c.* MnmG, (E) *E.c.* MnmG + Nb_MnmG_1, (F) *A.a.* MnmG. Although MnmG from *A. aeolicus* is mainly monomeric and only partially present as a dimer, we focused our analysis on the dimer peak, as MnmG from *E. coli* is clearly dimeric (see main text).

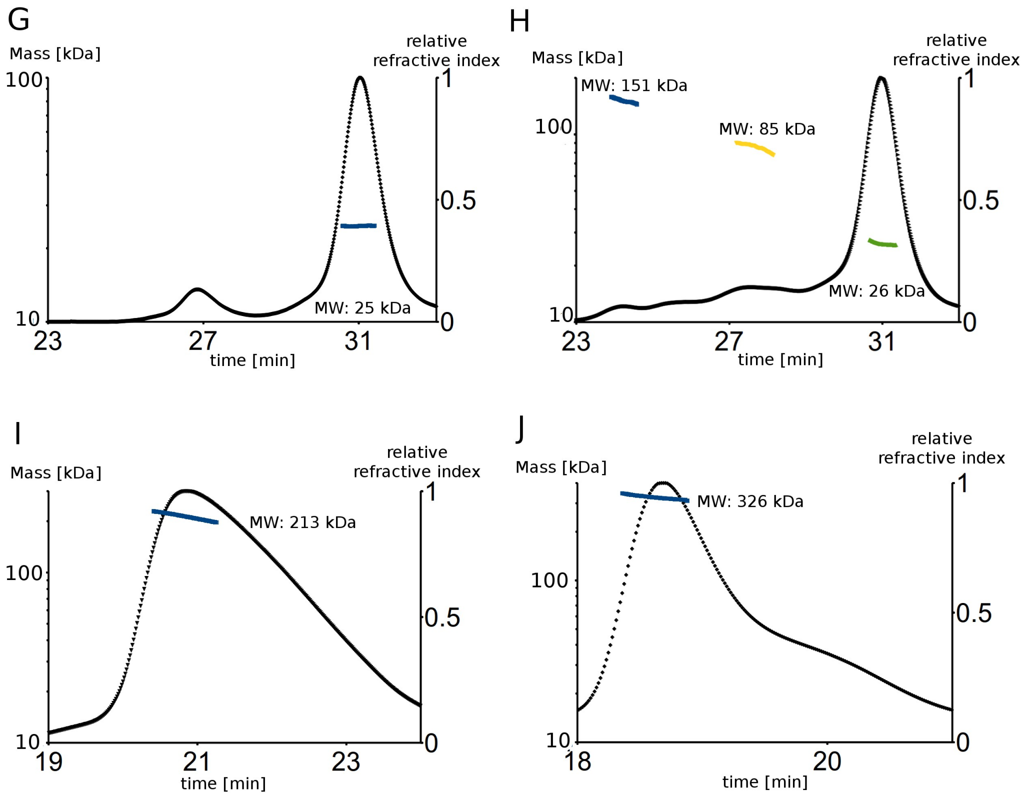

Figure S2 (continued): SEC-MALS data of (G) *A.a.* tRNA(Lys), (H) *A.a.* MnmG + tRNA(Lys), (I) *E.c.* MnmEG, (J) *E.c.* MnmEG-GDP-AlFx.

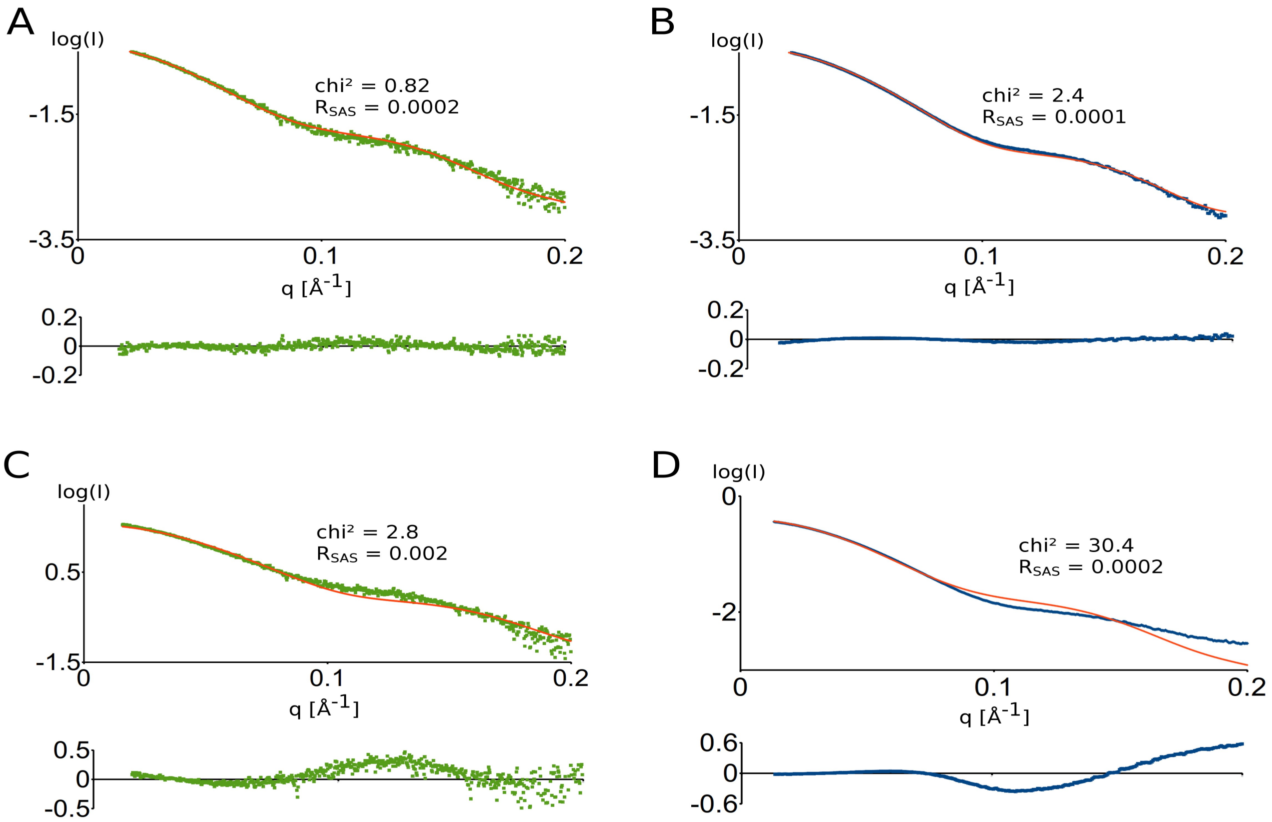

Figure S3: Comparisons of the quality of fit of the MnmE models in the "open" and "closed" states (see figure 1) against the experimental SAXS data in either the absence or presence of GDP-AlFx . The residuals are depicted below the fitting curves. (A) Fit of the open MnmE model against the data collected from MnmE in the nucleotide free state, (B) fit of the closed MnmE model against the data collected from MnmE in the GDP-AlFx bound state, (C) fit of the closed MnmE model against the data collected from MnmE in the nucleotide free state, (D) fit of the open MnmE model against the data collected from MnmE in the GDP-AlFx bound state.

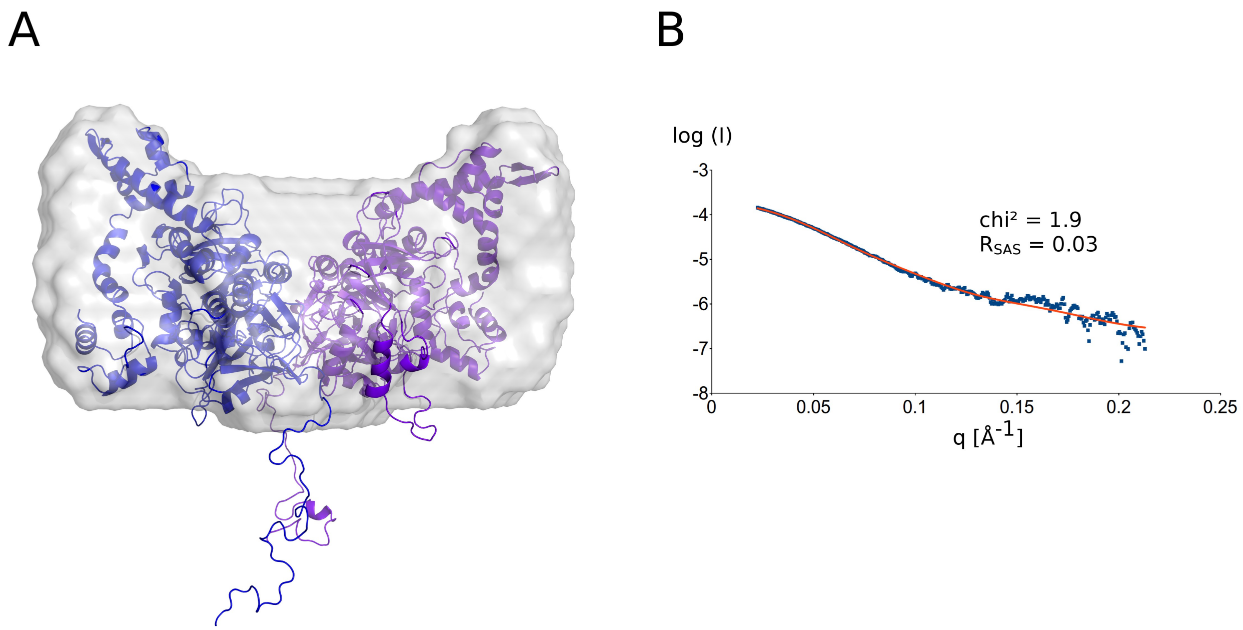

Figure S4: (A) Crystal structure of *A. aeolicus* MnmG (with modelled his tags and missing residues) superposed on the *ab initio* shape (gray) as obtained by Dammif. (B) The good fit of the experimental (blue) to the theoretical (red) scattering curve obtained by Crysol supports the model.

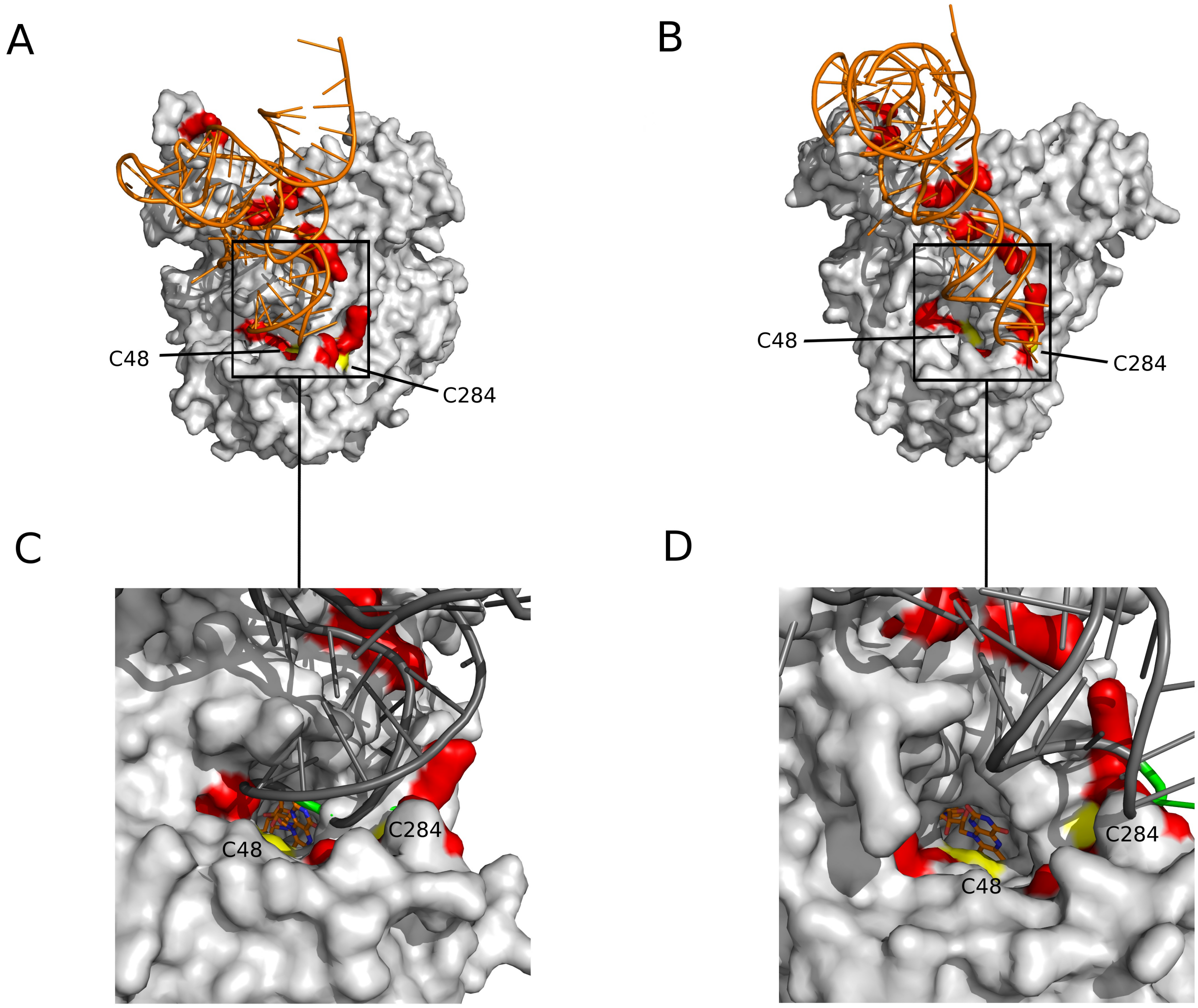

Figure S5: Comparison of the two MnmG-tRNA docking models that score equally well in Crysol. (A) Preferred and (B) alternative model of the MnmG-tRNA interaction; (C) and (D) give a close-up view of the respective (FAD-binding) active sites. Based on the known interacting residues (red), the catalytic cysteines (C48 and C284, yellow) and the location of the FAD co-factor (shown as stick model) with respect to the tRNA wobble nucleotide (green), the first model is preferred.

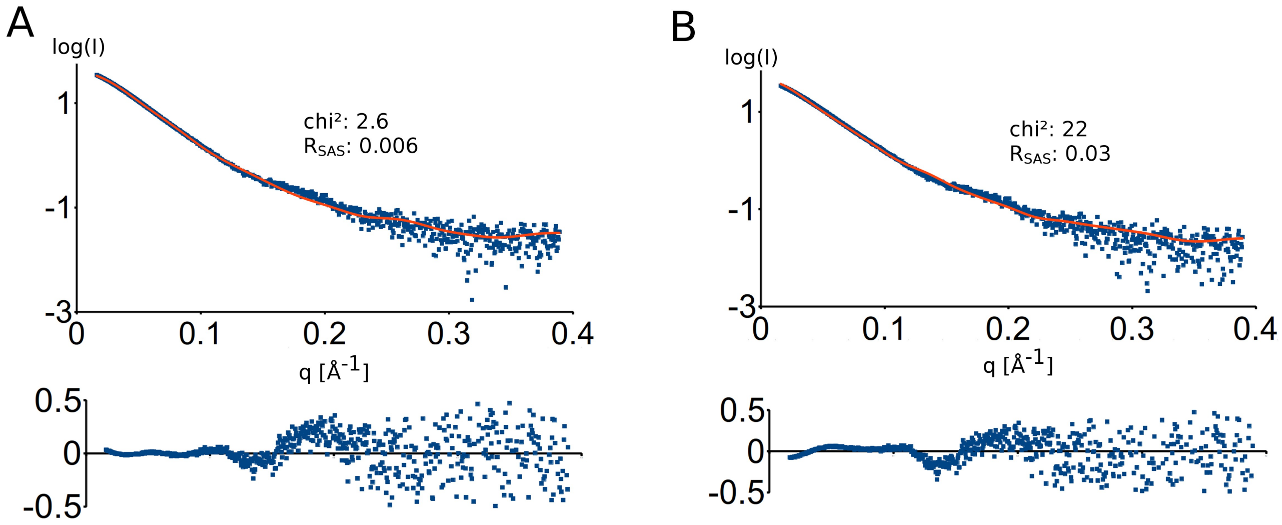

Figure S6: Comparison of the SAXS data fitting of the *A.a.* MnmG-tRNA complex using (A) only one tRNA or (B) two tRNAs. The residuals are depicted below the fits.

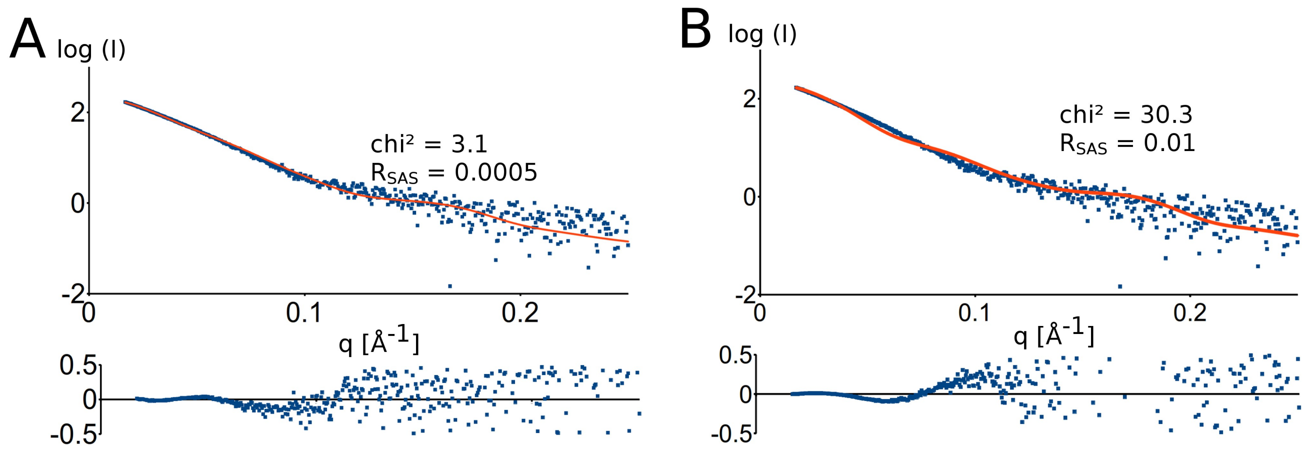

Figure S7: Comparison of the SAXS data fitting of the *E.c.* MnmEG complex using (A) the new model as proposed in this manuscript and (B) the model as previously proposed (Meyer, S., Scrima, A., Versées, W. and Wittinghofer, A. (2008) Crystal structures of the conserved tRNA-modifying enzyme GidA: implications for its interaction with MnmE and substrate. *J. Mol. Biol.*, **380**, 532–547). The residuals are depicted below the fits.

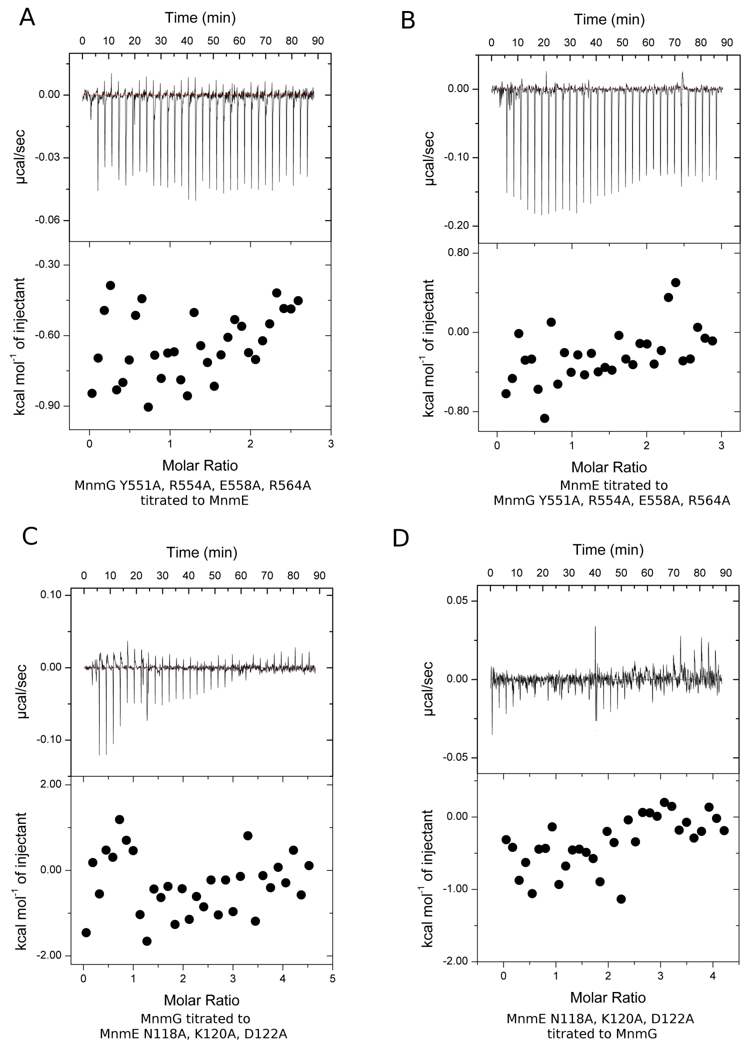

Figure S8: ITC data of the titration of (A) MnmG(Y551A, R554A,E558A,R564A) to MnmE, (B) MnmE to MnmG(Y551A, R554A,E558A,R564A), (C) MnmG to MnmE(N118A,K129A,D122A) and (D) MnmE(N118A,K129A,D122A) to MnmG in the presence of 1 mM FAD.

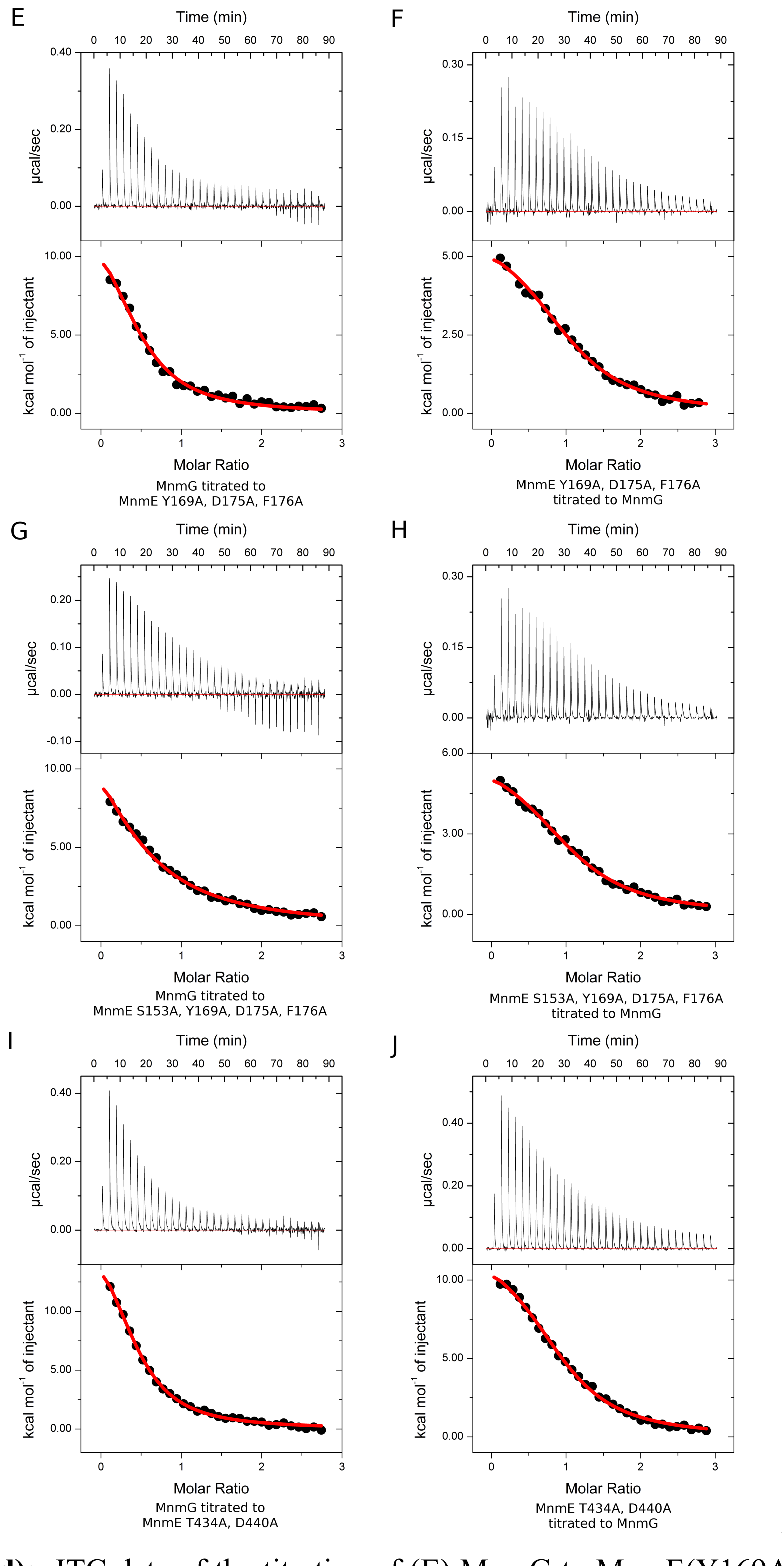

Figure S8 (continued): ITC data of the titration of (E) MnmG to MnmE(Y169A,D175A,F176A), (F) MnmE(Y169A,D175A,F176A) to MnmG, (G) MnmG to MnmE(S153A,Y169A,D175A,F176A), (H) MnmE(S153A,Y169A,D175A,F176A) to MnmG, (I) MnmG to MnmE(T434A,D440A) and (J) MnmE(T434A,D440A) to MnmG in the presence of 1 mM FAD.

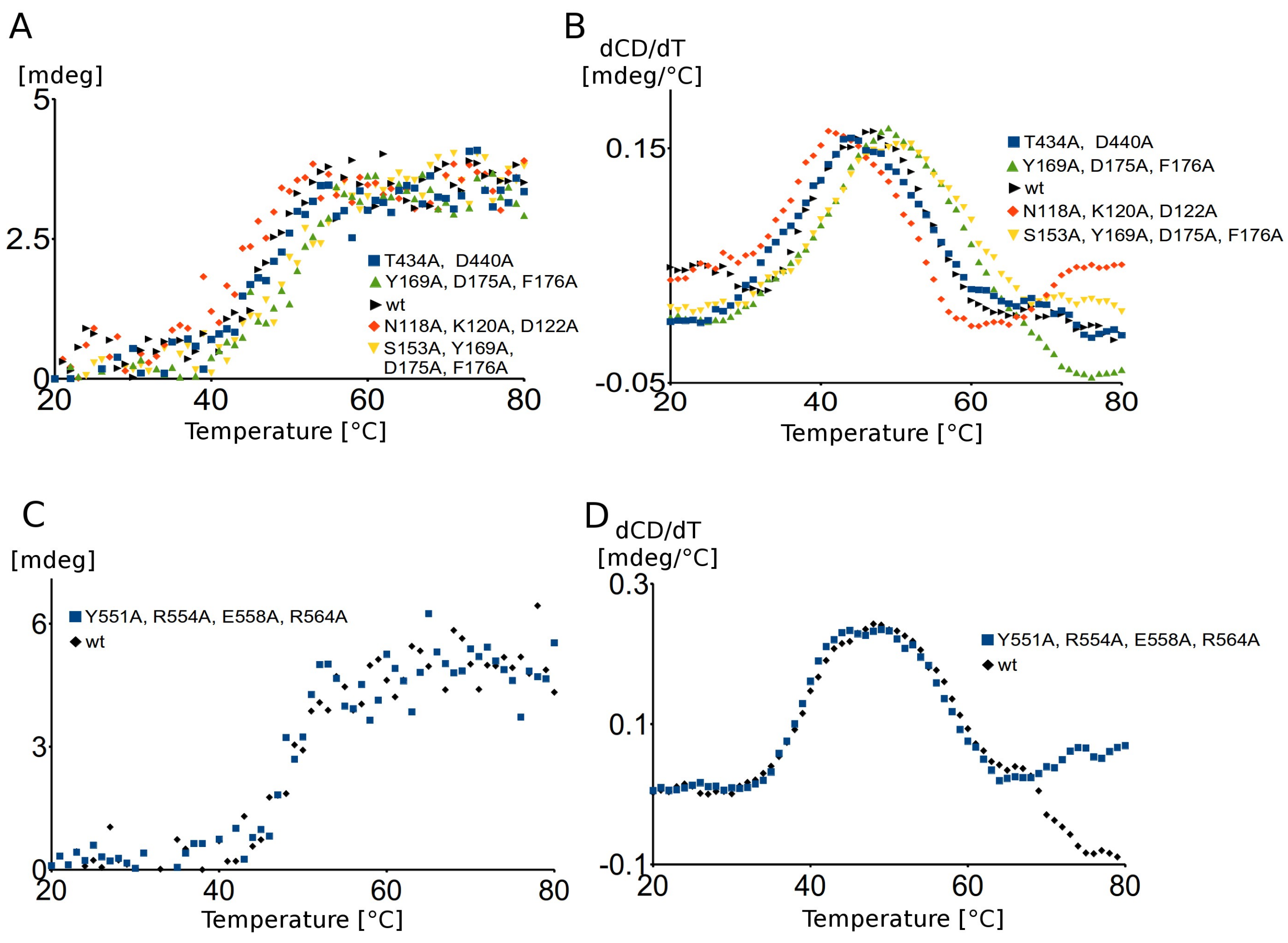

E

	T_M [°C]
**MnmE wt**	47
**MnmE T434A, D440A**	44
**MnmE S153A, Y169A, D175A, F176A**	50
**MnmE Y169A, D175A, F176A**	49
**MnmE N118A, K120A, D122A**	41

	T_M [°C]
**MnmG wt**	48
**MnmG Y551A, R554A, E558A, R564A**	49

Figure S9: Thermal unfolding curves as obtained for the MnmE and MnmG variants using circular dichroism. (A) Melting curves of the MnmE variants and (B) the first derivative. (C) Melting curves of the MnmG variants and (D) the first derivative. (E) Summary of the melting temperatures (T_M) of the different mutants indicate that the mutations do not significantly affect the stability of MnmE and MnmG.

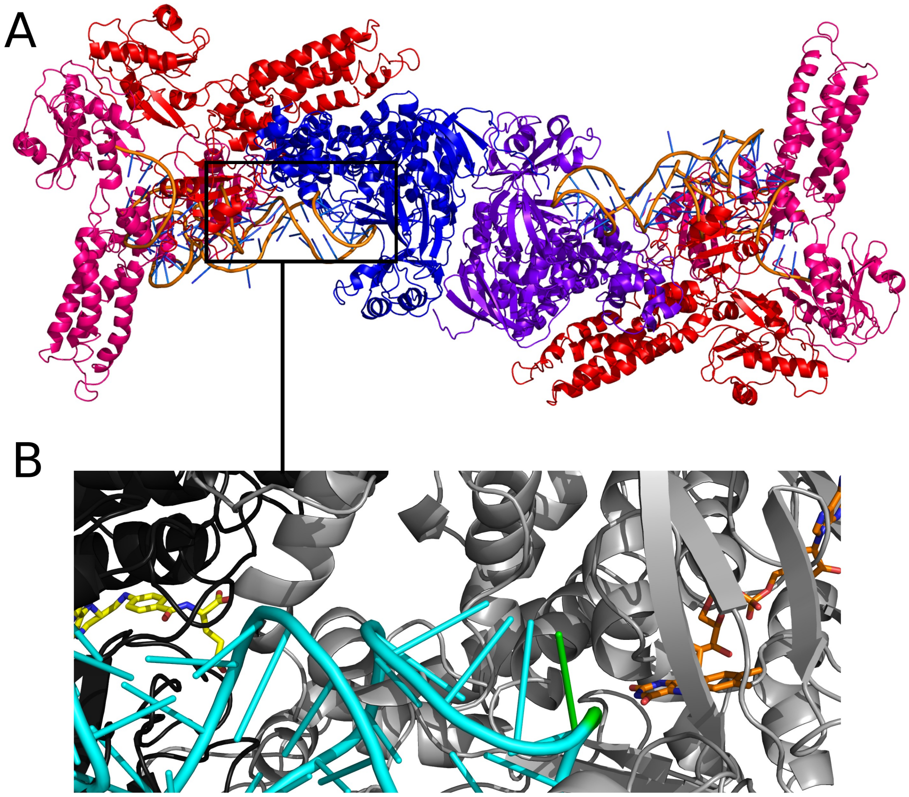

Figure S10: (A) Model of the MnmE-MnmG-tRNA complex as obtained by superposition of the MnmG-tRNA model onto the MnmEG-GDP-AlFx model. Additional conformational changes in the α-helical domains of MnmE upon GTP binding could relieve the sterical clashes that are present between tRNA and MnmE (B) A zoom into the active site shows, that the wobble nucleotide (green) is bound between the FAD co-factor (orange) coming from MnmG and the MTHF co-factor (yellow) provided by MnmE.

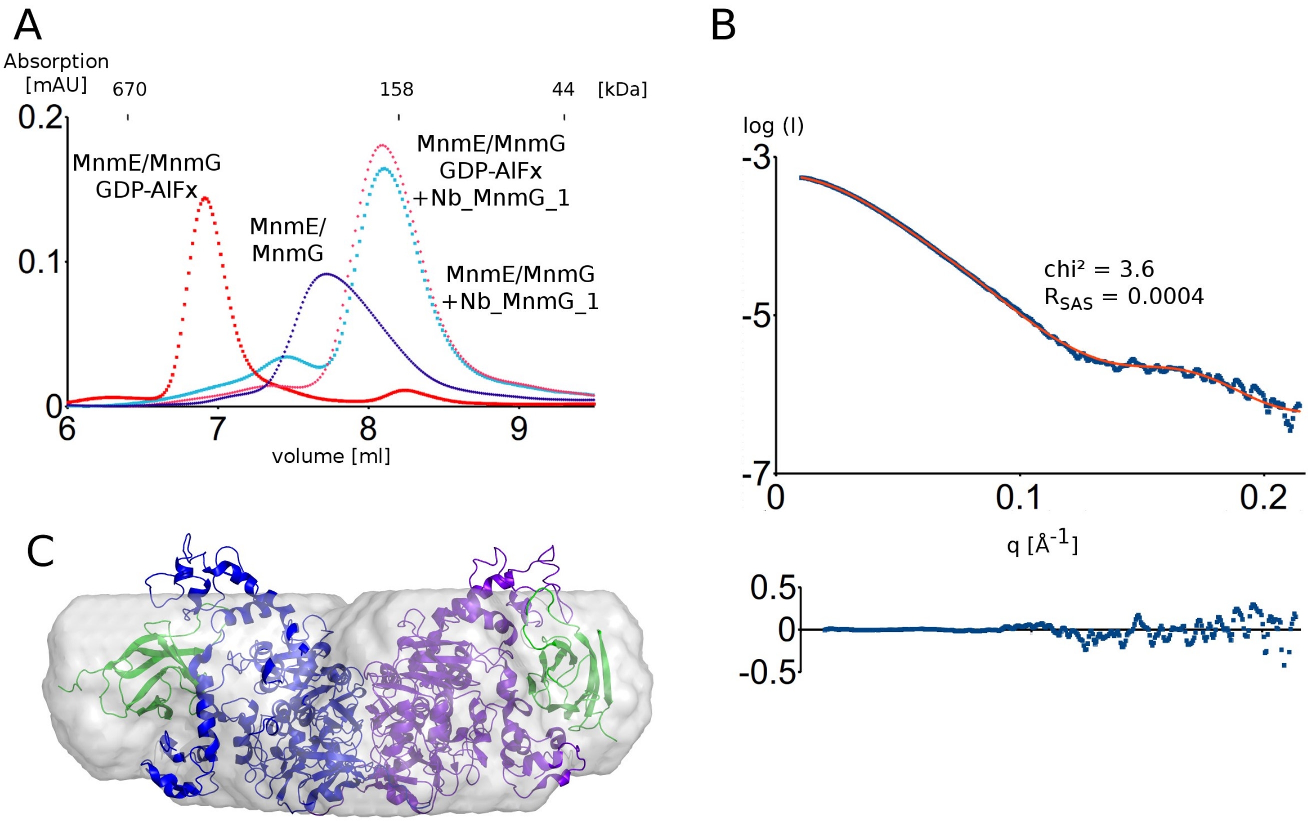

Figure S11: (A) Nb_MnmG_1 binding to MnmG disrupts the MnmEG complex in the α2β2 and α4β2 form according to gel filtration experiment. (B) The theoretical scattering curve (red) of the MnmG-Nb_MnmG_1 complex agrees well with the experimental scattering curve (blue). The residuals are depicted below the fit. (C) The model of MnmG (blue and purple) in complex with Nb_MnmG_1 (green) superposed on the *ab initio* shape as obtained by Dammif (gray).

A B

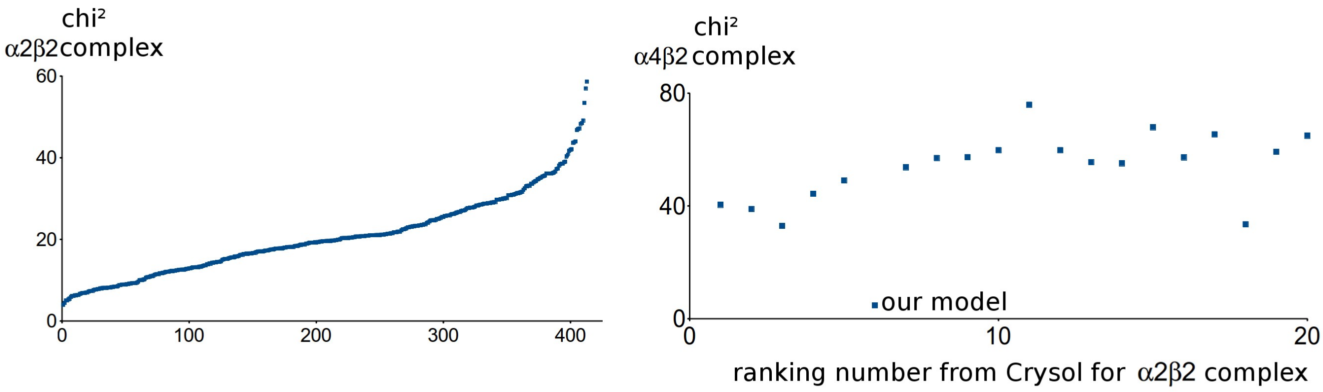

C

Docking Rank	Crysol Rank	chi² α2β2 complex	chi² α4β2 complex
277	1	3.92	40.4
2	2	4.41	38.9
53	3	5.02	32.9
159	4	5.06	44.3
288	5	5.34	49.0
8	6	5.48	4.7
49	7	5.90	53.7
156	8	6.10	57.0
206	9	6.10	57.3
51	10	6.30	59.8
138	11	6.30	75.9
173	12	6.35	59.8
186	13	6.35	55.5
100	14	6.66	55.1
126	15	6.71	67.9
201	16	6.81	57.2
191	17	6.86	65.4
36	18	6.92	33.5
302	19	6.97	59.2
14	20	7.02	64.9

Figure S12: (A) Ranking of all α2β2 MnmEG docking models using Crysol. (B) Crysol scores of the hypothetical α4β2 MnmEG models, resulting from the top 20 ranked α2β2 docking models. Only our selected model fits to the experimental SAXS data. (C) Summarizing table of the chi² values from crysol for the top20 ranked α2β2 docking models and the chi² value of the corresponding α4β2 MnmEG complexes. Note that the chi² values given in this table concern the models before molecular dynamics optimization.

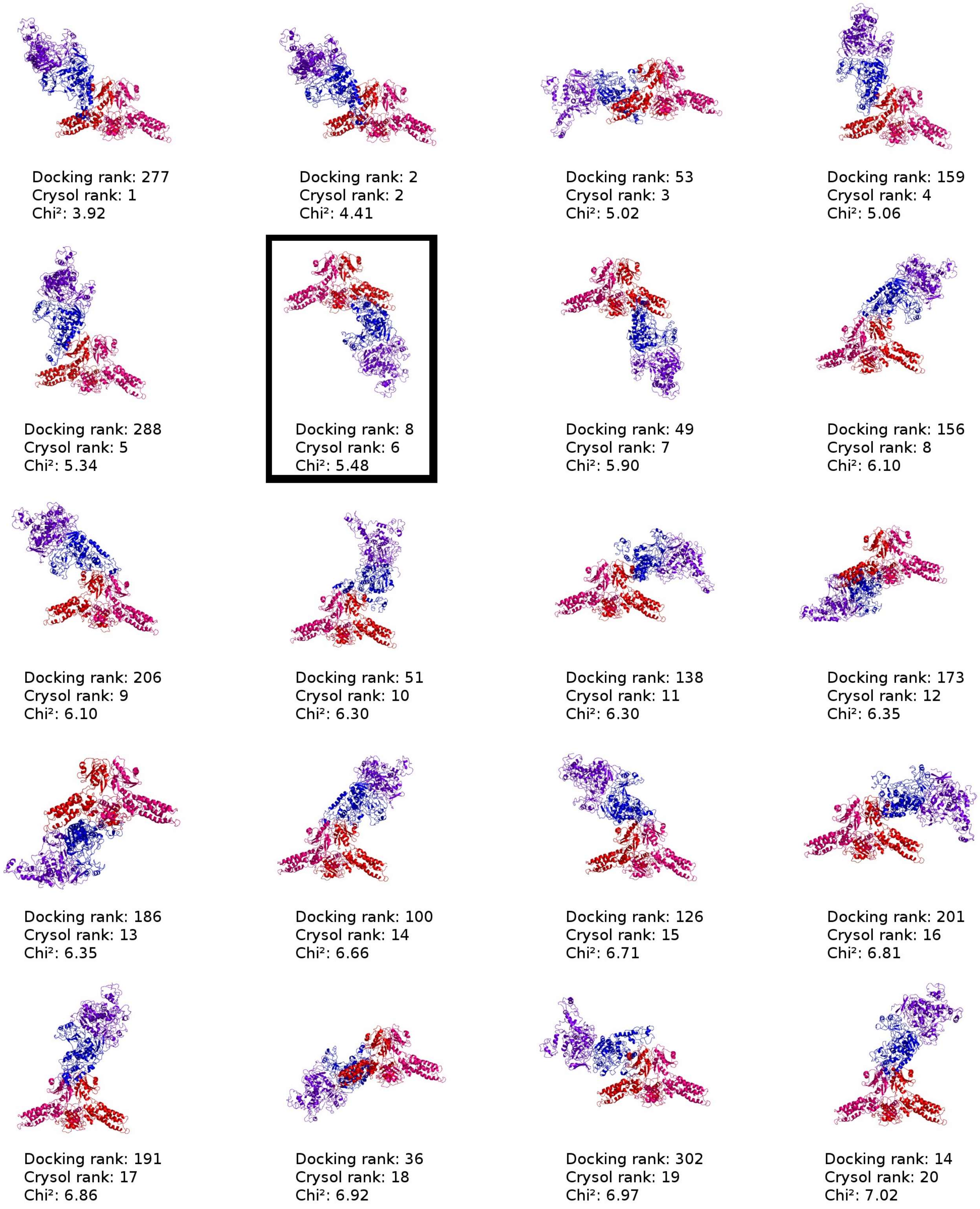

Figure S13: The top 20 of CRYSOL ranked docking models. The highlighted model represents our selected model.

Table S1: Summary of the statistics for the SAXS analyses.

	chi^2	R_{SAS}	MW_{SAXS} [kDa]	Rg_{exp} [Å]	Rg_{model} [Å]	Vc_{exp} [Å²]	Vc_{model} [Å²]	Qr_{exp} [Å³]	Qr_{model} [Å³]
E.c. MnmE open	0.82	0.0002	104	38.6 ± 0.9	38.1	706	711	12913	13268
E.c. MnmE closed	2.4	0.0001	121	37.2 ± 0.7	37	745	751	14908	15243
E.c. MnmE GppNHp	1.8	0.0002	109	37.4 ± 0.3	37.3	708	697	13403	13439
A.a. MnmG	1.9	0.03	168	42.3 ± 1.5	40.2	935	888	20667	19616
A.a. MnmG + tRNA	2.8	0.006	*	45.3 ± 0.8	42	800	779	14128	14449
E.c. MnmEG	3.1	0.005	207	52.3 ± 1.4	51.2	1144	1136	25561	25230
E.c. MnmEG GDP-AlFx	4.7	0.00007	338	67.4 ± 1.3	67.2	1675	1688	41626	42401
E.c. MnmG-Nb_MnmG_1	3.6	0.0004	184	43.3 ± 0.4	42.6	992	983	22727	22683

$R_{SAS} = (Rg_{exp}-Rg_{model})^2/(Rg_{exp})^2+(Vc_{exp}-Vc_{model})^2/(Vc_{exp})^2$

*) The experimental molecular weight of the tRNA-protein complex cannot be calculated since the reference parameters needed for this calculations do not exist for RNA-protein complexes (Rambo, R.P. and Tainer, J.A. (2013) Accurate assessment of mass, models and resolution by small-angle scattering. *Nature*, **496**, 477–481).

Table S2: Comparison of experimental molecular weights derived from SAXS and SEC-MALS, with the calculated molecular weight of the protein and complexes described in this paper.

	MW_{calc} [kDa]	MW_{SAXS} [kDa]	MW_{MALS} [kDa]
E.c. MnmE open	103	104	100 ± 5
E.c. MnmE closed	103	121	97 ± 5
E.c. MnmE GppNHp	103	109	98 ± 5
A.a. MnmG	150	168	130 ± 7
A.a. MnmG + tRNA	174	*	151 ± 10
E.c. MnmEG	243	207	213 ± 13
E.c. MnmEG GDP-AlFx	346	338	326 ± 26
E.c. MnmG-Nb_MnmG_1	173	184	170 ± 9
